# Targeted mutagenesis of *FATTY ACID ELONGASE 1* entails near complete elimination of very long chain fatty acids in the seeds of camelina cultivar Ligena

**DOI:** 10.1007/s42994-025-00238-z

**Published:** 2025-09-30

**Authors:** Barno Ruzimurodovna Rezaeva, Amélie A. Kelly, Martin Fulda, Ingrid Otto, Iris Hoffie, Sindy Chamas, Ivo Feussner, Jochen Kumlehn

**Affiliations:** 1 Plant Reproductive Biology, Leibniz Institute of Plant Genetics and Crop Plant Research (IPK) Gatersleben, Corrensstraße 3, 06466 Seeland, Germany; 2 Albrecht-Von-Haller Institute for Plant Sciences and Göttingen Center for Molecular Biosciences (GZMB), Department of Plant Biochemistry, University of Göttingen, Justus-Von-Liebig-Weg 11, Göttingen, Germany; 3 Research Centre for Horticultural Crops, Kühnhäuser Straße 101, Erfurt, Germany

**Keywords:** Adventitious shoots, Immature embryo, Targeted mutagenesis, Very-long-chain fatty acids, Erucic acid

## Abstract

Genome editing has the potential to enhance yield and quality traits of crops. However, standard genetic transformation methods are not always applicable to modern germplasm. To tackle this challenge in the widely cultivated variety Ligena of the oilseed crop camelina (*Camelina sativa* (L.) Crantz), an only recently established principle of adventitious shoot formation from immature zygotic embryos was employed to further improve its fatty acid profile. In this approach, the three subgenomic homeologs of the *FATTY ACID ELONGASE 1* (*FAE1*) gene were subjected to targeted mutagenesis. To pre-validate the Cas9-interacting, target motif-specific guide (g)RNAs, a robust protoplast-based DNA transfection method was established. This assay demonstrated that the preselected gRNAs were capable of eliciting mutations across all three camelina *FAE1* homeologs. Likewise, targeted mutagenesis was successful at the whole-plant level. Triple-homozygous *fae1* knockout mutants were identified amongst a segregating generation M_3_ family. Gas chromatography of lipid extracts from M_4_ seeds revealed a significant increase in all unsaturated C18 fatty acids including the particularly valuable α-linolenic acid. This was accompanied by a near elimination of the C20 and C22 very long-chain fatty acids including the nutritionally problematic erucic acid. Altogether, we have developed camelina elite lines with two significantly improved properties of high relevance for a health-promoting human nutrition.

**Supplementary Information:**

The online version contains supplementary material available at 10.1007/s42994-025-00238-z.

## Introduction

Plant oils are an essential component of our daily life. They can be used directly as food and indirectly in the production of industrial goods. Commodity oilseed crops such as soybean, oil palm, cottonseed, rapeseed, and sunflower provide 75% of the current global production of vegetable oils (Mondor and Hernández‐Álvarez 2022). Camelina is also an oilseed crop and contains 15 to 20 different fatty acids, with more than 50% of the total oil content being composed of the long-chain polyunsaturated fatty acids (lcPUFAs) linoleic acid (18:2^∆9,12^, n-6) and α-linolenic acid (18:3^∆9,12,15^, n-3). In comparison to most other common oilseed crops, camelina seeds contain a significantly higher proportion of α-linolenic acid (35%) along with a high content of tocopherols (Putnam and Müller 1993; Righini et al. 2016), which renders it an outstanding resource for the provision of healthy food and for the generation of renewable starting materials for the chemical industry. On the other hand, disadvantages of camelina include its small seed size and low oil content of seeds as well as the lower seed yield under common agricultural conditions as compared with canola. In addition, sinapines and glucosinolates contained in the seeds can reduce the quality of products. The oxidative stability of camelina oil is higher than that of linseed oil, which is mainly due to the somewhat lower content of PUFAs and the significantly higher content of tocopherols (Mondor and Hernández‐Álvarez 2022). However, the still limited shelf life of the oil remains a disadvantage compared to the more common food oils from canola, sunflower, and olive seeds.

Fatty acid profiles of oilseeds have been adapted in different ways to the specific requirements of the various utilization principles, i.e., by crossing in spontaneously occurred mutations, induced random mutagenesis, transgenesis, and genome editing. Erucic acid is a quality-limiting fatty acid that has been associated with myocardial lipidosis, among other things (Chien et al. 1983), and is, therefore, undesirable in human food and animal feed. Erucic acid-reduced breeding lines were already reported for rapeseed 60 years ago (Stefansson and Hougen 1964), a feature that has long been considered standard for this major oilseed crop. The high content of the lcPUFAs linoleic acid and α-linolenic acid, on the other hand, is a decisive and well-known quality aspect for the value of camelina oil in terms of a healthy diet. Camelina is not only a valuable oilseed crop in its own right, but can serve as an excellent model plant for genetic and metabolic engineering in modern biotechnology (Yuan and Li 2020). Its close genetic relationship with *Arabidopsis* might be a reason for its amenability to genetic transformation via floral dipping (Lu and Kang 2008). During the last years, a number of studies have been devoted to the validation of gene function and the improvement of various seed quality features of camelina by means of RNA-guided Cas9 endonuclease (Jiang et al. 2017; Morineau et al. 2017; Aznar-Moreno and Durrett 2017; Ozseyhan et al. 2018; Lee et al. 2021; Hölzl et al. 2023).

To speed up target gene-specific mutagenesis in camelina, a two-step workflow was developed and the *FAE1* homeologs were targeted, while screening for the loss of very long-chain fatty acids (VLCFAs, C ≥ 20) is straightforward and well established for seed lipid extracts. To functionally test the suitability of in silico pre-selected target motifs (and their cognate gRNAs), first a protoplast assay was established and employed. Upon the production of *cas9*/gRNA transgenic plants and identification of primary mutants, repeated selfing and progeny analysis yielded two M_3_ lines with different mutation patterns in which all three *FAE1* homeologs present in camelina were homozygous dysfunctional. Analyses of the fatty acid profiles of seeds from these lines showed that, compared to the cv. Ligena parental line, they had higher levels of unsaturated C18 fatty acids, while the fractions of VLCFAs, including erucic acid that is considered problematic for human nutrition, were reduced to hardly detectable levels.

## Results

### Identification of the *FAE1* homeologs in camelina cultivar Ligena

*FAE1* homeologs were amplified from genomic DNA from camelina cv. Ligena using homeoallele-independent primers. Sequencing with homeoallele-specific primers revealed clones for each of the three homeologs. All three *FAE1* homeologs consist of only one exon and show high sequence similarity to each other.

### Cas9-induced mutagenesis of *FAE1* in camelina protoplasts

**Fig. 1:**
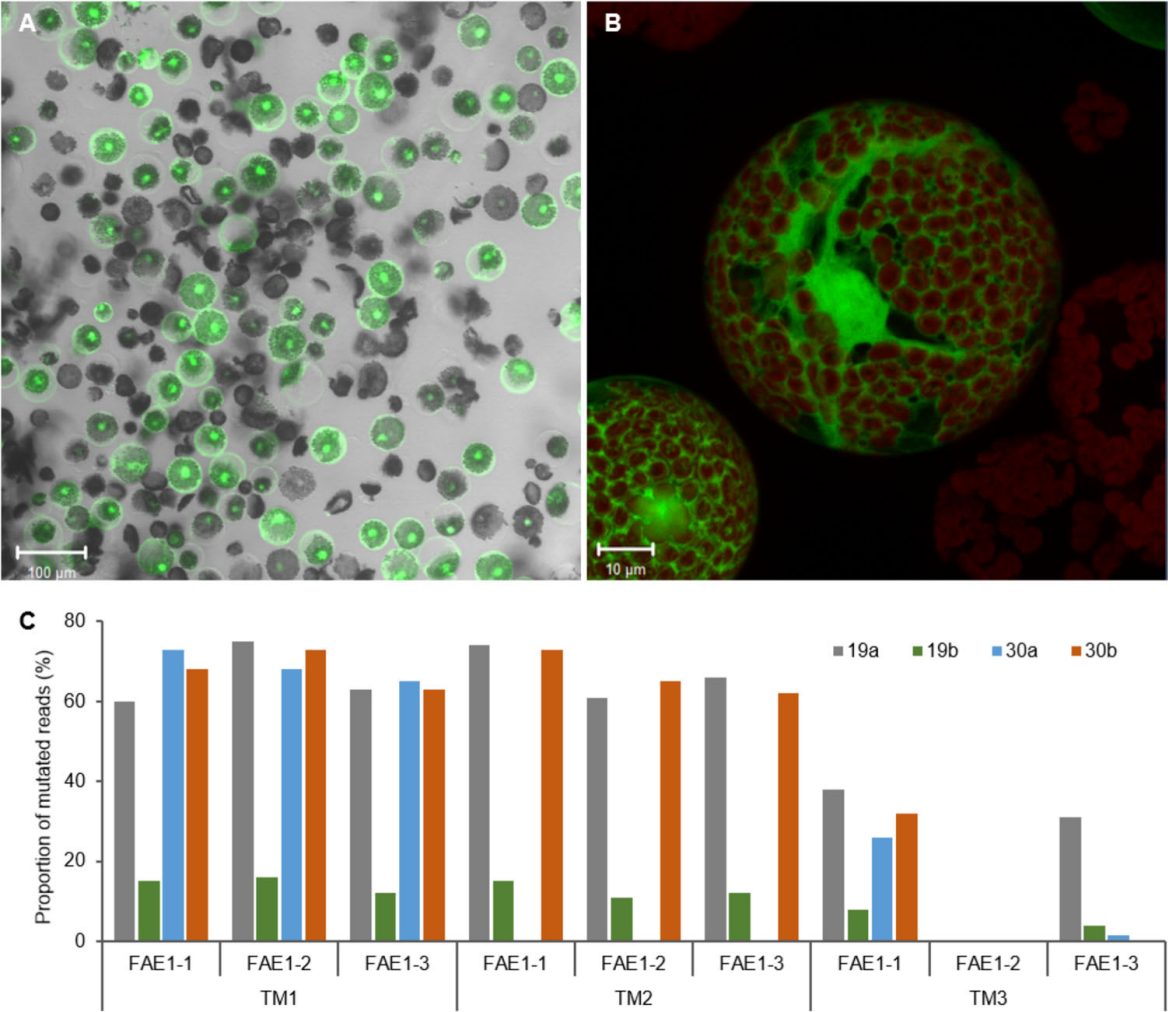
Analysis of mutagenesis efficiency.** A** Representative image showing isolated, GFP-positive mesophyll protoplasts two days after PEG-mediated DNA transfer. **B** Individual protoplasts at higher magnification. The fluorescence serves as a positive control for both protoplast viability and transfection. The frames shown were generated by merging bright-field and confocal-microscopic images. From such protoplasts, genomic target regions were amplified and sequenced to pre-validate the *FAE1*-specific gRNA/*cas9* construct. **C** Mutagenesis efficiencies of target motifs (TM1, 2 and 3) in the three *FAE1* homeologs of M_1_ plants 19a, 19b, 30a, and 30b from experiment CB13, as represented by proportions of mutated amplicon deep-sequencing reads related to total reads

Three target motifs (TMs) were selected to mutagenize *FAE1*, with all of them being present in all three homeologs. To assess the mutagenesis efficiency of the cognate gRNAs, the intermediate construct pBR37 that contains expression units for *cas9* and the three target motif-specific gRNAs was subjected to a camelina protoplast-based assay involving PEG-mediated DNA transfer. To estimate the transfection efficiency, a *GFP*-carrying plasmid was transferred in a simultaneously conducted approach. 18 h after transfection, the proportion of GFP-positive protoplasts was ca. 63% (Fig. 1A,B). Upon the extraction of genomic DNA and amplification of the target region, individual sequence reads could be attributed to the three individual homeologs due to the presence of homeoallele-specific single-nucleotide polymorphisms (Fig. S1). All three TMs proved amenable to mutagenesis across the three homeologs. Several hundred reads were obtained for each TM. The proportion of mutated sequence reads related to the total read numbers (including those derived from the > 30% non-transgenic protoplasts still present after transfection) was as high as 15.3% for TM2 (Table 1).

**Table 1:** Protoplast-based pre-validation of target motifs. The protoplast transformation efficiency of ca. 63%, as estimated according to a simultaneously conducted approach using GFP, is not considered in the data presented here

Target motifs (incl. PAM)		No. of total reads per homeolog			No., % and patterns of mutated reads			Total mutation frequency
		*FAE1*-*1*	*FAE1*-*2*	*FAE1*-*3*	*FAE1-1*	*FAE1-2*	*FAE1-3*	
1	CCACTTCTATTCCCATCTCCAAC	1745	1949	2283	282 (14%)	328 (16.0%)	248 (10.0%)	13.3%
	most occurring patterns				-TC, -CTA	+ T, + A	+ C, + T	
2	GAGACAGAGCAGGTAATCATCGG	1556	1385	1410	294 (19.0%)	156 (10.1%)	285 (17.0%)	15.3%
	most occurring patterns				-GTAAT, -TCATC	+ A, + T	+ A, + T	
3	GCAAAAGGAAGGATGAAGAAAGG	3503	2313	2587	265 (7.0%)	18 (0.8%)	80 (3.0%)	3.6%
	most occurring patterns				-A, -11, -14	-A, -11	-A, -11	

An equally successful result (13.3%) was obtained for TM1. However, TM3 proved less efficiently mutated (3%) than TM1 and TM2. The results of amplicon deep-sequencing revealed a predominant presence of small deletions of 1 to 5 base pairs or insertions of a single base pair (C, T or A) at the expected Cas9 cleavage sites of TM1 and TM2. In TM3, however, somewhat larger deletions of 11 and 14 bp also occurred, as illustrated in Fig. S2.

### Generation of *fae1* mutant plants

#### Screening M_1_ plants for mutations

The target-specified gRNAs proved effective in camelina protoplasts and were thus also used for targeted mutagenesis of *FAE1* at the whole-plant level. To this end, four transformation experiments were conducted, each using 60 immature embryos of cv. Ligena for *Agrobacterium*-mediated DNA transfer. In every experiment, different *Agrobacterium* optical densities (OD_600_ values of 0.3, 0.6, and 0.9) were applied using 20 explants each. Primary transgenic plants were created with an overall efficiency of 3.8% based on the number of explants. In total, nine transgenic plants were obtained (Table 2). Inoculation with agrobacterial ODs of 0.3 and 0.9 produced transformants in almost all experiments, whereas treatments with OD 0.6 did not yield a single transgenic plant (Table S1).

**Table 2:** Summary of the camelina transformation experiments using construct pBR38

Experiment code	No. of explants	*cas9*^+^	*mCHERRY*^+^	No. of primary transgenic plants (%)*
CB7	60	1	1	1 (1.7)
CB8	60	2	2	2 (3.3)
CB11	60	2	2	2 (3.3)
CB13	60	4	4	4 (6.7)

^*^The percentages refer to the numbers of explants used to reflect the overall efficiency of the procedure

Approximately 40 resulting regenerants were analyzed per experiment by *mCHERRY*- and *cas9-*specific PCR assays for the presence of T-DNA and by screening the target regions for mutations either by Sanger sequencing and/or deep sequencing using homeolog-specific primers (Tables S2, S3 and S4). Detailed genetic analyses were conducted on five primary transformants. Preliminary amplicon Sanger sequencing revealed that plant CB7/1 had heterozygous mutations, with evidence of a large inversion of its DNA sequence between the cleavage sites of TM1 and TM3 in homeolog *FAE1-1*. For homeolog *FAE1-2*, the mutation type could not be determined, since the PCR primer binding sites were apparently affected owing to a particularly large modification. In contrast, mutations in the allele *FAE1-3* included a single base deletion (T) and a single base insertion (T) at the Cas9 cleavage sites of TM1 and TM2, respectively. The results of amplicon deep sequencing of transformants obtained in experiment CB13 are shown in Table 3. As indicated by smaller case letters, M_1_ plants CB13/19a and 19b derive from the same immature embryo explant. The same applies for plants CB13/30a and 30b. Fig. S3 illustrates the sequence variants with the highest frequency of mutation reads in plants derived from experiment CB13. TM1 and TM2 proved mutated in all three *FAE1*-*1*, *2*, and *3* homeologs across all four primary mutants, except TM2 of plant 30a. By contrast, TM3 was less effectively mutated, with mutations being observed only in *FAE1*-*1* and *3* (Fig. 1C and Fig. S3). The proportions of mutation reads were consistently over 50% in both TM1 and TM2 across all three homeologs in primary mutants 19a and 30b (Fig. 1C). The mutation patterns in the target motifs of primary transgenic plants obtained in experiment CB13 involved single-base deletions and insertions as well as somewhat larger deletions up to 14 base pairs in size.

**Table 3:** Progeny analysis and identification of triple homozygous *fae1* mutant lines

M_1_ plant code	Analyzed M_2_ progeny	*cas9*^+^	Number of M_2_ mutant plants	Number of triple homozygous M_2_ plants
CB7/1	14	9	9	2
CB13/19a^*^	5	5	5	0
CB13/19b	5	0	5	0
CB13/30a	5	5	5	0
CB13/30b	5	5	5	0

^*^Smaller case letters are given to specify differently mutated sister lines derived from the same embryo explant

While plants 19a and 19b that derived from the same explant showed only a few identical mutations, they were different in most of the TMs. Although the sister plants 30a and 30b carried mostly identical mutations, they differed in at least one target motif of each *FAE1* homeolog. Mutation patterns and frequencies are presented in detail in Fig. S3. All mutant alleles are likely to be dysfunctional owing to the multiple modifications and translational reading frame shifts. Differences in mutagenesis efficiencies obtained in the three TMs in plants were found to be similar to those observed in the protoplast assay (Table 1 and Fig. 1C). Most strikingly, TM3 exhibited a markedly low editing amenability in both plant cells and protoplasts. In addition, the mutation patterns that had predominantly occurred in protoplasts were also observed in the genomes of whole-plant samples (Fig. S2 and S3).

#### Screening of M_2_ plants for mutations

Table 4 presents the results regarding transgenicity and targeted mutations in M_2_ progeny of five primary mutant plants. A total of 14 M_2_ plants were produced from M_1_ transformant CB7/1. Of these, nine were screened for mutations in TM1 and TM2 using homeolog-specific primers. However, owing to the high similarity between the three *FAE1* homeologs, these allele-specific primers were found to bind to some extent to all three homeologs, which is why no clear differential amplification was possible. Three CB7/1 descendants (CB7/1–13, 17, and 18) were then analyzed using generic primers binding to all three homeologs and covering all three target motifs by 1460-bp long amplicons. The mutation frequencies of M_1_ plants derived from experiment CB13 are given in Fig. S3, and amplicon deep-sequencing data of five M_2_ siblings derived from CB13/19a are shown in Fig. S1 and S4. Each TM was successfully amplified and sequenced in all tested M_2_ plants using the generic primers (Table S2). Taken together, mutations were detected in all 34 M_2_ individuals analyzed (Table 3).

**Table 4:**
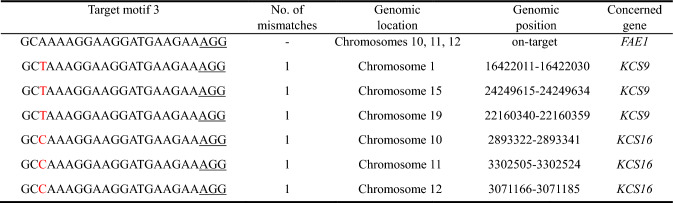
Polymorphisms between the addressed on-targets and potential off-targets of the *cas9*/gRNA constructs used in this study. PAMs are underlined and mismatches between off-target site and gRNA are shown in red

Fig. S5 displays the results of the amplicon Sanger sequencing of M_2_ plants CB7/1–13, 17, and 18 using generic homeolog-unspecific primers (Table S2: GP400 and GP405). Nonetheless, all reads were unambiguously assigned to their individual homeologs, taking advantage of single-nucleotide polymorphisms present in the genomic sequences. Intriguingly, two distinct types of novel potential junctions for the inversion of the target region between TM1 and TM3, and TM2 and TM3 in *FAE1*-*1* and *FAE1*-*2* were identified.

In *FAE1-1* of plants CB7/1–13 and 18, a 1192-bp fragment from TM1 to TM3 was inverted, which was accompanied by a loss of 5 bp in TM1 and another deletion of 2 bp in TM2 at their Cas9 cleavage sites. Another inversion of 833 bp was found between TM2 and TM3 in *FAE1*-2 along with a base insertion (T) at the cleavage site of TM2 in plants CB7/1–13, 17, and 18. Notably, the amplification of the *FAE1*-*3* target region was straightforward compared to those of *FAE1*-*1* and *FAE1*-*2*. TA cloning of PCR products indicated that the CB7/1–17 and 18 triple mutant plants were homozygous. The mutations in homeolog *FAE1*-*3* were predominantly small insertions and deletions, ranging from one to three base pairs for CB7/1–13, 17, and 18 (Fig. S4). CB7/1–13 was bi-allelic as to *FAE1*-*1*, with two types of mutation detected, namely a big deletion between TM1 and TM2 and an inversion of a sequence stretch between TM1 and TM3. Unfortunately, the mutation patterns of the *FAE1*-*1* and *FAE1*-*2* homeologs could not be identified for plants CB7/1–15 and 16.

To perform a mutation screen on plants obtained in the experiment CB13, five M_2_ descendants were randomly selected from each of the four primary mutants. The results of amplicon deep-sequencing demonstrated that TMs 1, 2, and 3 were mutated in all three *FAE1* homeologs in the majority of CB13-derived M_2_ plants. However, none of these plants proved to be a triple homozygous *fae1* mutant (Fig. S1 and S5).

### Compromizing FAE1 function decreases VLCFA in M_3_ and M_4_ seeds

**Fig. 2:**
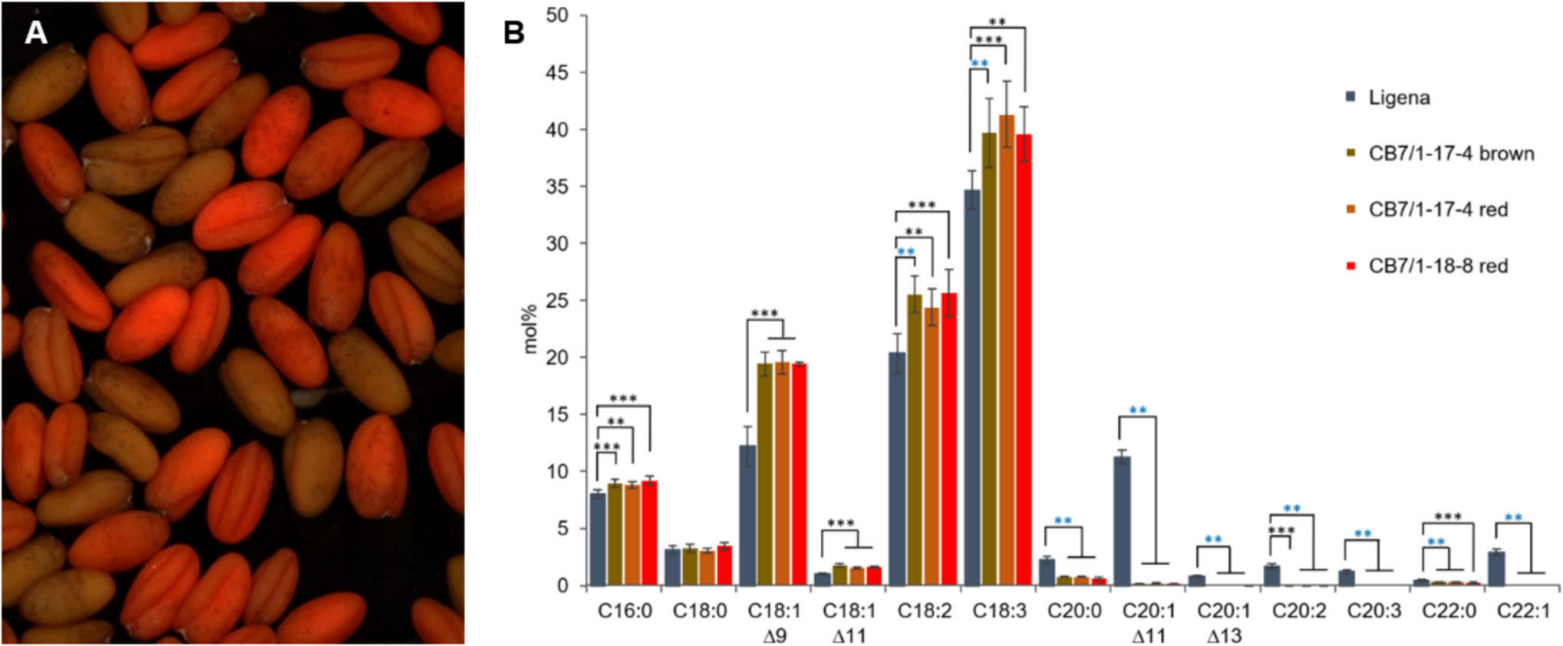
Analysis of the fatty acid profile of seeds. **A** Segregation of red (transgenic, *mCHERRY*-expressing) and brown (non-transgenic) M_3_ seeds. **B** Relative amounts of major fatty acids (mol%) as determined by GC-FID analysis in seeds of cv. Ligena (wild-type) and M_4_ seeds of M_3_ plants derived from the primary (*cas9*/gRNA/*mCHERRY*) transgenic plant CB7/1. Error bars denote standard deviation of six samples each containing 20 seeds. For CB7/1–17-4, two sorts of samples were analyzed: one containing mCHERRY-fluorescent seeds (transgenic) and the other containing non-fluorescent (non-transgenic) segregants. Black asterisks indicate significant differences (** for *P* ≤ 0.01, *** for *P* ≤ 0.001) according to ANOVA followed by Tukey test, and blue ones stand for respective significance levels according to Kruskal–Wallis analysis followed by Student–Newman–Keuls method, with the latter procedure being applied if data had not passed normal distribution and/or equal variance tests

To investigate the effect of the observed mutations in *FAE1* on the fatty acid profile, five M_2_ plants (CB7/1–13, 15, 16, 17, and 18) were selected and grown for seed formation. 15–20 either red fluorescing (*mCHERRY*-expressing) or brown (segregating individuals without T-DNA but still mutant) seeds were selected per M_2_ plant by UV illumination (Fig. 2A) and screened for their fatty acid composition by GC-FID of FAMEs.

Compared with the wild-type, all mutant lines contained drastically reduced levels of VLCFAs, i.e. gondoic acid (20:1^∆11^) and erucic acid (22:1^∆13^) in M_3_ seeds, with no substantial difference being observed in the VLCFA content between the red and brown seeds. Moreover, most lines showed a clear increase of oleic acid (18:1^∆9^) as well as polyunsaturated FAs (18:2^∆9,12^ and 18:3^∆9,12,15^) which are the substrates of *FAE1* and direct precursors of eicosenoic acids (Fig. S6). This result suggests that the observed triple *FAE1* mutation caused almost 100% reduction in the VLCFA content compared to the wild-type.

For a subsequent screen, M_4_ seeds (involving both brown and red samples) derived from M_3_ plants CB7/1–17-4 and CB7/1–18-8 were analyzed. GC-FID analysis of the total FA composition confirmed a nearly 100% loss of all unsaturated VLCFAs across all samples. As observed for the previous generation, this was the case for both red and brown seeds and accompanied by a significant increase in most LCFAs (Fig. 2B). Despite the drastic changes in the composition of individual FAs, total lipid content in the mutant seeds remained unchanged compared to the wild-type, as shown in Fig. S7. This indicates that the overall capacity for lipid biosynthesis in the seeds was unaffected by the *fae1* mutations. M4 plants were grown under greenhouse conditions directly next to wild-type plants. No obvious deviations in growth type, vigor and fertility were observed. The germination capability of the seeds also seemed to be unaffected across all mutant generations.

### Off-target analysis

It is important to make sure that the gRNA 5’-ends align precisely with their cognate target motif, while avoiding potential matches with other sites within the genome. In the case of TM1 and TM2, no perfectly matching off-target was identified due to the presence of several mismatches. The off-targets carrying some mismatches where not followed up on any further. However, two potential off-target sites were identified that differed only in a single mismatch compared with TM3 (Table 4).

One candidate off-target motif was identified within the *3-KETOACYL-CoA SYNTHASE 9* (*KCS9*) across all three homeologs (Chromosomes 1, 15 and 19), while *KCS16* contained the second candidate site. *KCS* genes are members of the *FAE1*-*LIKE* multigene family that encode enzymes responsible for synthesizing a diverse range of VLCFAs such as C22 and C24 in higher plants (James Jr et al. 1995). These off-target motifs exhibited 22 out of 23 base pair identity, and perfectly matched with the PAM along with the 17 bps upstream of it. Previous research has demonstrated that nucleotides in position -18 upstream of the PAM do not substantially contribute to the specificity of gRNA-target motif interaction (Jiang et al. 2013). Therefore, these two off-targets were analyzed for the occurrence of Cas9-elicited mutations in the triple homozygous *fae1* mutant line CB7/1–18.

Using seven M3 plants, homeolog-unspecific amplification of these off-target regions within *CsKCS9* and *CsKCS16* followed by deep-sequencing revealed both wild-type and mutated reads in case of *CsKCS9*, whereas no mutant reads were seen for *CsKCS16* (Fig. S8). The assignment of wild-type and mutated reads to the three *CsKCS9* homeologs suggests that none of the plants analyzed was triple homozygous mutated in this off-target. Therefore, at least residual gene function can be anticipated.

## Discussion

The use of RNA-guided Cas endonucleases represents a highly effective approach to the validation of gene function and to crop improvement. This comparatively young technology is still rapidly evolving. In this study, we provide a further example of how important traits of crop plants can be improved by taking advantage of the precision and unprecedented effectivity of genome editing.

### Validation of *cas9*/gRNA constructs via protoplast transfection

The in silico prediction of suitable target motifs for Cas9-elicited mutagenesis is associated with some uncertainty (Naim et al. 2020). Mesophyll protoplast assays based on transient expression of Cas endonuclease and gRNA(s) followed by amplification and sequencing of the target regions have proved useful for testing the functionality of preselected target motifs (and their cognate gRNAs) much faster than is possible at the whole-plant level (Koeppel et al. 2019). Meaningful examples of this are available for the crops rice and wheat (Shan et al. 2014), tomato (Čermák et al. 2015), rapeseed (Lin et al. 2018) and barley (Hoffie et al. 2021), among others. However, such assays are not yet available for many less explored plant species, which has so far also been the case for camelina. Consequently, we have developed an efficient protoplast isolation and transfection method for targeted mutagenesis in this species to our very best knowledge for the first time. Its application involved the validation of the activity of a *cas9*/gRNA construct targeting three pre-selected TMs present in the *FAE1* gene of the cv. Ligena that enjoys a high market share in central Europe.

For two of the Cas9 target motifs addressed in our protoplast experiments, mutation rates of about 13 and 15% were achieved, while for a third target motif, the proportion of mutated deep-sequencing reads was only 3% (Table 1). However, considering that effective DNA transfer was obtained in around 65% of protoplasts (Fig. 1A), as determined by a control experiment using a *GFP* construct, the stated mutation rates represent an underestimation of around 35%. Of note, the mutagenesis efficiency achieved in any of the target motifs proved to be similar across the three *FAE1* homeologs (Table 1). In related *Brassicaceae* crop species, protoplast transfection systems have been established previously (Wang et al. 2015; Sahab et al. 2019; Sidorov et al. 2022). The mutation rates achieved in various studies with different *cas9*/gRNA constructs were as high as 75% for rapeseed and 57% for broccoli (Lin et al. 2018). In the case of cabbage (*Brassica oleracea*) and Chinese cabbage (*B. rapa*), up to 2 and 24% respectively, were achieved (Murovec et al. 2018). In the present study, the pre-validation of the target motifs proved to be sufficiently informative and predictive for the mutations subsequently achieved at the whole-plant level, both in terms of the relative efficiency of the target motifs to each other as well as regarding the mutation patterns obtained (Fig. S1, S2, S3 and S5).

### Camelina transformation using immature embryos facilitates genome editing of the current cultivar Ligena

The present study represents the first use case of recently established methods of adventitious shoot formation from camelina immature embryos and the utilization of these explant types for *Agrobacterium*-mediated genetic transformation (Rezaeva et al. 2024, 2025). The dipping method previously used for camelina transformation was, in our own experience, limited by a considerable genotype dependency, which could be overcome by the above-mentioned methods thanks to the particularly high totipotency of hypocotyl tissue of the immature embryos used. This principle is, therefore, also considered promising for further applications in camelina and, after methodological adaptation, maybe even for other members of the *Brassicaceae* family.

### Effective mutagenesis of the target gene by simultaneous expression of multiple gRNAs

It is a challenging endeavor to address multiple target sites simultaneously at different genomic positions. Camelina is an allohexaploid (amphidiploid) species with a complex genome including three related subgenomes that often exhibit high sequence identity (Hutcheon et al. 2010). Consequently, the majority of genes comprise three pairs of homeologs, which results in six copies per gene in the G1 phase of the cell cycle and even 12 in G2. To cope with the presence of multiple homeologs, a particularly useful approach is to utilize multiple guide RNAs targeting conserved regions shared by all homeologs of a given target gene (Ghidoli et al. 2023). The functional pre-validation of the gRNAs in combination with the use of three gRNAs per target gene proved successful in the present study in that mutations were present in all three homeologous alleles in all transgenic plants generated with only a single exception. Accordingly, the production of homozygous triple-knockout lines has already been achieved by producing the first selfing generation using just a few primary transformants. In camelina, gRNAs expressed simultaneously were thus far used in only one previous study, in which the production of completely homozygous mutants was also readily achieved (Hölzl et al. 2023).

### Challenges in screening for mutations in polyploids

Genetic analysis of polyploids is a complex process (Abe et al. 2019). This complexity increases when multiple gRNAs are used to cope with gene redundancy and to achieve effective editing. In our study, we detected comparatively large inversions by simultaneously amplifying all three TMs and performing TA cloning of the PCR product using a homeolog-unspecific approach. One of the mutations observed in the *FAE1-1* homeolog was an inversion of the entire fragment between the cleavage sites of the first and third TMs (Fig. S4). Additionally, an inversion occurred in the second homeolog between TM2 and TM3. Such inversions have also been reported in other organisms using Cas9-mediated genome editing, including rice and maize (Lu et al. 2021; Schwartz et al. 2020), *Arabidopsis* (Schmidt et al. 2020; Zhang et al. 2018), tobacco (Gao et al. 2015), silkworm (Liu et al. 2014), and zebrafish (Xiao et al. 2013).

Amplicon deep sequencing is a straightforward approach for identifying small mutations, such as insertions or deletions, at the cleavage site. However, this approach can be insufficient for detecting larger genomic rearrangements due to the limited amplicon size (typically 200 to 300 bp). Our results suggest that TA cloning of PCR products with generic (homeolog-unspecific) primers, coupled with Sanger sequencing of long amplicons (at best amplifying all TMs simultaneously), is the most reliable method for detecting previously unknown mutations, even though it is somewhat labor-intensive.

### Homozygous triple-mutated plants were obtained in offspring of primary transgenics

In the present study, most mutations observed in the primary mutant plants proved inheritable to M_2_ plants. Two of the fourteen M_2_ lines from the primary transgenic plant CB7/1 showed triple homozygous mutations (siblings 17 and 18), while plant 13 was also a full knockout involving a bi-allelic mutation in *FAE1-1* and homozygous mutations both in *FAE1*-*2* and *FAE1-3*. These results indicate that it is indeed possible to obtain triple homozygous camelina mutants by just one selfing step. Additionally, the progeny of M_2_ plants were subjected to Sanger sequencing, which confirmed triple homozygosity of M_3_ plants derived from plant CB7/1–18, with the mutation pattern in M_3_ being identical to that of the ancestor (Fig. S4).

### Off-target mutations in the *KCS9* gene are unlikely to interfere with the fatty acid profile of seed oil

The off-target analysis of *fae1* knockout mutants revealed that mutations in the *KCS9* gene also occurred in these plants. The mutated off-target only differs from the on-target in a single base position. While the plants examined to this end were homozygous mutant in all *FAE1* homeologs, they did not show a complete knockout of *KCS9*, as in all cases the functional wild-type sequence was present in at least one *KCS9* homeolog (Fig. S8). *KCS9* is involved in the elongation of C22 to C24 fatty acids that are essential precursors for the biosynthesis of cuticular waxes, aliphatic suberin, and membrane lipids, including sphingolipids and phospholipids (Kim et al. 2013). This gene is expressed in various organs and tissues, including roots, leaves, stems, epidermis, silique walls, sepals, the upper portion of the styles, and seed coats, but not in developing embryos. Its preferential expression in stem epidermal cells (Suh et al. 2005) agrees with the assumption that *KCS9* is involved in cuticular wax biosynthesis rather than in the formation of seed oil (Millar et al. 1999; Fiebig et al. 2000; Hooker et al. 2002; Lee et al. 2009).

### Targeting *FAE1* resulted in much reduced accumulation of VLCFAs

The *FAE1-LIKE* multigene family (comprising 21 putative *KCS* genes in *Arabidopsis*), including *FAE1* encodes enzymes responsible for synthesizing a diverse range of VLCFAs in higher plants (James Jr et al. 1995). It has been shown previously that the sequences of the 1518 bp-long camelina *FAE1* homeologs (*FAE1-1*, *-2*, *-3*) are highly similar to one another (i.e. greater than 96% identity in the reference genome line Cs32) (Hutcheon et al. 2010). Moreover, such as camelina, *Arabidopsis* seeds contain high amounts of VLCFAs. Gondoic acid (20:1^∆11^) and its much less abundant isomer paullinic acid (20:1^Δ13^) are found exclusively as components of triacylglycerols in seed oil where they together amount to about 10 to 15 mol% of the total FAs in camelina and ca. 19 mol% in *Arabidopsis*. They are both synthesized by *FAE1* that produces gondoic acid by the elongation of 18:1^∆9^ to 20:1^∆11^ as well as paullinic acid by elongating 18:1^Δ11^ to 20:1^Δ13^. Paullinic acid amounts to only 2 mol% in whole *Arabidopsis* seeds, but is specifically enriched in the endosperm (Bryant et al. 2016). Mutations of *FAE1* in *Arabidopsis* led to decreased levels of VLCFAs in seeds (Kunst et al. 1992). In the camelina experimental model accession Suneson, targeting *FAE1* using RNA-guided Cas9 endonuclease resulted in a reduction of VLCFAs to less than 2% of the total FAs, compared to more than 22% in the wild-type, with a corresponding increase in C18 unsaturated FAs (Ozseyhan et al. 2018). In the present study, we evaluated the effect of triple *FAE1* knockout mutations in the current camelina cultivar Ligena by analyzing the fatty acid composition of selected M_3_ and M_4_ seeds of five differently mutated M_2_ descendants originating from the primary *fae1* mutant CB7/1 using gas chromatography, and compared the results with the fatty acid composition of wild-type seeds. While these M_3_ and M_4_ seeds were homozygous mutant in the target gene, they segregated into transgenic and non-transgenic individuals, which was visualized by the presence and absence of an mCHERRY signal under UV illumination (Fig. 2A). GC analysis of pooled red (mCHERRY) or brown (T-DNA-free) M_3_ and M_4_ seeds (Fig. 2B) showed a strong reduction in VLCFAs to hardly detectable levels (Fig. S6). This result confirms that FAE1 is responsible for most of the VLCFA accumulation in camelina seeds.

In this context, the question arises whether the elimination of VLCPUFAs such as gondoic and erucic acid could lead to impaired plant performance, as these fatty acids can fulfill diverse physiological functions in both generative and vegetative tissues. So far, however, we have no data on this beyond the general observation mentioned in the results section that differences between mutants and wild-type in terms of plant development and fertility were at least not obvious.

As expected, the loss of the VLCFAs was associated with concomitant increases of most of the C18 long-chain fatty acids. Here, a slight fluctuation between samples can be most likely attributed to the growth conditions, since it is known that the fatty acid profile of seeds is strongly influenced by the prevailing water, temperature, and light conditions. The observation that comparable outcomes were obtained across M_3_ and M_4_ pools derived from events carrying different mutation patterns as well as for both transgenic and non-transgenic M_3_ and M_4_ seed subpopulations supports not only the idea that the detected mutations are stable and inherited though the germline, but also provides solid evidence that *FAE1* dysfunction results in a reduction of VLCFAs to hardly detectable levels in camelina seeds. In this study, the elimination of erucic acid is achieved in non-transgenic background of the widely grown camelina cultivar Ligena, which improves the opportunity to produce oil with unprecedented quality and value for human nutrition.

## Materials and methods

### Plant material and growth conditions

The camelina cultivar (cv.) Ligena (Deutsche Saatveredelung, Lippstadt-Bremen, Germany) was used in this study. The seeds were grown in glasshouse chambers under natural daylight supplemented with 16 h illumination by sodium high-pressure lamps providing an additional light intensity of 500 μmol m^−2^ s^−1^. The day/night temperature regime was adjusted to 20/18 °C, and the relative humidity was maintained at 65%. The plants were regularly watered with a 1% solution of fertilizer Hakaphos Blau (15% nitrogen, 10% phosphorus, 15% potassium; Compo Expert, Germany).

M_4_ and corresponding Ligena seeds were obtained from randomized plants grown under long-day conditions, 16 h of light / 8 h of darkness, at a constant temperature of 22 °C.

### Cloning of the *FAE1* knockout construct

**Fig. 3:**
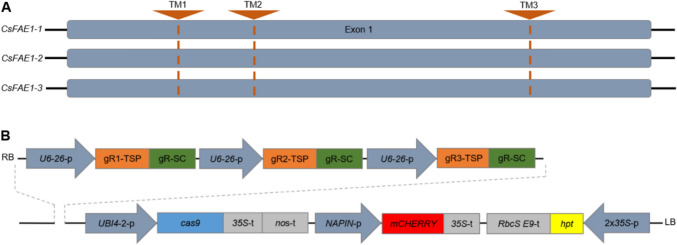
Cas9 target motif (TM) selection and vector construction.** A** Gene structure of *FAE1* homeoalleles and location of target motifs. **B** Schematic representation of the binary vector (pBR38) used for plant transformation. The modular vector contains the *cas9* endonuclease expression unit with *UBIQUITIN 4–2* promoter (*UBI4*-*2*-p) from parsley and the two combined Cauliflower Mosaic Virus (*35S*-t) and *nopaline synthase* (*nos*-t) terminators. The selection marker *mCHERRY* is expressed under control of the *NAPIN* promoter (*NAPIN*-p) and *35S*-t, *hygromycin phosphotransferase* (*hpt*) from *E. coli* is under control of the doubled-enhanced *CaMV35S* promoter (2 × *35S*-p). The expression of the single guide RNAs each consisting of a 20 bp target-specific part (gR-TSP, orange boxes) and the gRNA scaffold (gR-SC, green boxes) is under control of the polymerase III-processed *U6-26* promoter (*U6-26*-p) from *Arabidopsis*. LB, left T-DNA border; RB, right T-DNA border

A pre-selection of candidate target motifs (TMs) was conducted using the DESKGEN online platform (Doench et al. 2016). Further selection criteria for TMs were applied according to Koeppel et al. (2019) under consideration that the TMs are identically present in all three homeologs of *FAE1* (Fig. 3A).

The candidate gRNAs’ secondary structures were then modeled using the RNAfold WebServer (Gruber et al. 2008) after adding the gRNA scaffold to the 20 bp of the target-specific 5'-end (N_20_GUUUUAGAGCUAGAAAUAGCAAGUUAAAAUAAGGCUAGUCCGUUAUCAACUUGAAAAAGUGGCACCGAGUCGGUGCUUUU). Three most suitable TMs were finally selected from the single *FAE1* exon (1518 bp) (Supplementary sequence S1). Figure 3A displays the localization and Table 5 the sequences of the TMs. To generate an *FAE1* construct, the modular CasCADE vector system based on hierarchical Golden Gate cloning was utilized, as described by Hoffie (2022). Briefly, forward and reverse DNA oligonucleotides (Table S5) were generated for gRNA containing overhangs for BbsI-based insertion into the gRNA module vectors pIK75 to pIK77 containing the *Arabidopsis U6* promoter (*U6*-*26*-p). The three individual gRNA module vectors were assembled using the backbone vector pIK61 to generate the gRNA assembly vector pBR36. This gRNA assembly was then combined with the *cas9* endonuclease expression unit (pBR20) and an *mCHERRY* reporter gene module to generate pBR21 (Table S6). The intermediate assembly vector pBR37 contained the three gRNA expression units, an *Arabidopsis* codon-optimized *cas9* under control of the parsley *UBIQUITIN* promoter 4–2 (*PcUBI4-2*-p), and monomeric *CHERRY* (*mCHERRY*) as visual reporter, expressed under control of the seed-specific *NAPIN* promoter from *Brassica napus*. Finally, the genome editing modules of pBR37 were cloned into the generic binary vector p6i-d35S-TE9 (DNA CLONING SERVICE e.K., Hamburg, Germany) via SfiI restriction and ligation. The binary vector pBR38 additionally contains the *hpt* gene controlled by a doubled-enhanced *CaMV35S* promoter for plant selection (Fig. 3B). The final vector was verified through Sanger sequencing and introduced into the hypervirulent LBA4404/pSB1 strain of *Agrobacterium tumefaciens* via electroporation.

**Table 5:** In silico predicted activity scores of target motifs (TM) and the modeled secondary structures of their cognate gRNAs used for targeted mutagenesis of *FAE1* in camelina

Target motifs	gRNA-specific part	PAM	Activity scores		2-D structure of gRNAs
			Wu CRISPR	Deskgen	
TM1	GTTGGAGATGGGAATAGAAG	TGG	87	48	
TM2	GAGACAGAGCAGGTAATCAT	CGG	98	55	
TM3	GCAAAAGGAAGGATGAAGAA	AGG	93	48	

### Isolation and PEG-mediated transfection of protoplasts

#### Protoplast isolation

The procedure outlined in Yoo et al. (2007) for *Arabidopsis* protoplast isolation was modified for application in camelina. Briefly, the plant material was cultivated in a greenhouse. Healthy two-week-old leaves were sliced into 30 × 30 mm pieces with a razor blade, ensuring that most cells were not ruptured (approximately 8 to 12 leaf strips were used). The cut leaf strips were immersed in a freshly prepared 10-mL enzyme solution containing cellulase and macerozyme (Table S7) to digest cell walls. The enzyme solution was first infiltrated for 30 min at 500 mbar in a vacuum desiccator and then, samples were incubated for 6 h at 24 °C in the dark. Following this, 50 mL of W5 buffer (Table S7) was added to proceed with the isolation of protoplasts. The suspension containing the released protoplasts was passed through a 70-μm nylon sieve into a 250-mL reaction flask and rinsed three times with 50 mL of W5. The protoplast suspension was subjected to centrifugation for 3 min at 60 g and room temperature. The supernatant was removed, and the protoplast pellets were resuspended in 500 μL of W5 before being transferred to a 7-mL tube. The protoplasts for transfection were then left on ice for at least 30 min to let them settle to the bottom of the tube. The supernatant was discarded and the protoplasts were re-suspended in 1 mL MMG (Table S7). Next, a representative sample of 20 μL of protoplast suspension was transferred to a Fuchs-Rosenthal counting chamber for observation and counting using a PrimoVert microscope manufactured by Carl Zeiss (Oberkochen, Germany).

#### PEG-mediated protoplast transfection

For transfection, 500,000 protoplasts (in approximately 100–200 μL) were mixed with 20 μL of DNA (total 20 μg) in a 2-mL reaction vessel, along with an equal amount of freshly prepared polyethylene glycol (PEG) solution (Table S7). The intermediate assembly vector pBR37 (detailed in Table S6) was used for transformation. In a parallel experiment, a synthetic *GREEN FLUORESCENT PROTEIN* gene (*GFP*)-containing construct (pGH429, Rezaeva et al. 2024) was used as a control to determine the percentage of transfected protoplasts. Protoplast transformation was conducted in three replicates for both constructs pBR37 and pGH429. The mixture of protoplasts, DNA and PEG was inverted and tapped to ensure proper mixing for transfection. Following a 30-min incubation period in the dark, 220 μL of W5 was added and gently swirled. The resulting protoplasts were left to stand for 3 min and centrifuged for another 3 min at 80 g at room temperature. After that, the protoplasts were suspended in 1 mL W5 and transferred to 12-mm dishes (Greiner BioOne Cellstar, Germany) that had been surface-treated with 0.1% bovine serum albumin (BSA) using a wide pipette tip. The protoplasts were incubated for 18 to 24 h at 24 °C and 28 °C in the dark to assess the impact of temperature on the mutation rate. Subsequently, images were captured to enumerate the number of protoplasts expressing *GFP* using an Axiovert 200 M fluorescence microscope (Carl Zeiss, Oberkochen, Germany) equipped with an Axiocam 506 color camera.

#### Analysis of targeted mutagenesis in protoplasts

After the incubation period following transfection, genomic DNA was extracted from the protoplasts. For this, the protoplast suspension was transferred into a wide-bottomed 2-mL reaction tube and filled with W5 buffer (Table S7). The tubes were then centrifuged at room temperature for 3 min at a rate of 16,000 *g*. Following centrifugation, the supernatant was completely removed. The pellet was resuspended in 300 µL TPS buffer (100 mM Tris–HCl, 10 mM EDTA, 1 M KCl pH 8.0). To break remaining cell walls, the tube was dragged over a 96 Eppendorf rack for 15 s and incubated at 50 °C for 3 min. After adding 300 µL of isopropanol (−20 °C), it was incubated again for 20 min at room temperature. Subsequently, the nucleic acids were precipitated by centrifugation at 16,000 *g* for 20 min. The pellet was then washed by mixing vigorously with 1 mL of 70% EtOH for 5 min. Finally, the dried pellet was dissolved in 50 µL of TE buffer (1 M Tris–HCl, 0.5 M EDTA, pH 8.0) containing RNase (10 mg/mL) and incubated for 1 h at 37 °C. The concentration of DNA was measured, and amplification was performed using allele-unspecific primers (Table S2) targeting 200–250 bp of the desired regions. The deep-sequencing of amplicons was performed using an Illumina MiSeq platform (Genewiz/Azenta). For deep-sequencing, the PCR mixture (50 µL) was purified using a column (Thermo Fisher Scientific) and its DNA content was measured by a NanoDrop device (Thermo Fisher Scientific). The DNA was normalized to 20 ng/µL and 10 different DNA samples of PCR amplicons were pooled to generate the sequencing sample for amplicon deep-sequencing. The raw data were analyzed using the Galaxy IPK server (https://galaxy.ipk-gatersleben.de/). The mutation efficiency of each replicate was determined by calculating the proportion of sequencing reads that displayed a mutation in relation to the total number of reads, including the wild-type ones.

### *Agrobacterium*-mediated camelina transformation

The genetic transformation of camelina was performed according to the protocol described by Rezaeva et al. (2024) using immature zygotic embryos. Briefly, immature zygotic embryos of spring camelina cv. Ligena (1.2–1.6 mm) were excised from harvested silicles at the medium-walking stick stage 12–14 days after pollination. Three days-old pre-cultured hypocotyls of immature embryos were pricked at 3 to 5 sites with a sterile hypodermic needle (0.3 × 25 mm; BD PrecisionGlide™, USA) under a binocular microscope (Stemi 2000, Zeiss, Germany). The injured explants were immediately transferred to liquid inoculation medium in a 6-well plate (CellStar, gainer Bio-One, Germany) to prevent desiccation. The liquid medium was then completely removed from the 6-well plate containing immature embryos and 3 mL of pBR38-containing *Agrobacterium* suspension (OD_600_ 0.3, 0.6 and 0.9) was added to each well. During the initial step of inoculation, the plate was placed in a desiccator and a vacuum of 450 mbar was applied for 3 min. The vacuum system consisted of a vacuum pump (Diaphragm Pump, VAUUBRAND, Germany) to which a desiccator was connected. After switching off the pump, air was slowly let into the desiccator (over a period of 60 s) to minimize tissue damage. The *Agrobacterium* suspension was removed after a 12-min incubation without vacuum, and the explants were briefly blotted onto filter disks. Afterwards, the infected immature embryos were co-cultivated in the dark at 21 °C for 48 h on pre-culture medium including 150 μM acetosyringone. Co-cultivation was followed by a selection period in the presence of Timentin (150 mg/L) to remove *Agrobacterium* and in the presence of hygromycin (50 mg/L) to facilitate the preferential development of transgenic cells and tissues. Plantlets were grown in a greenhouse until the formation of mature grains.

#### Amplification and sequencing of genomic target regions of mutants

Two week-old leaf samples were collected for DNA extraction using a phenol–chloroform-based protocol (Pallotta et al. 2000). The presence of T-DNA in regenerated plantlets was confirmed by PCR using specific primers for *cas9* and *hpt* (Table S4). Mutation screening was performed using allele-specific or -unspecific primers via polymerase chain reaction (PCR)-based amplification of the target regions (TMs 1, 2 and 3) (Table S2 and S3), followed by amplicon deep-sequencing based upon Illumina technology (conducted by Genewiz, Leipzig, Germany).

#### TA-cloning of PCR products

PCR was carried out with allele-unspecific primers (Table S2: GP400 and 405) and Phusion high–fidelity DNA polymerase. The resulting product was purified and cloned into pJet 1.2 (Thermo Scientific, Germany). Clones containing inserts were identified through colony PCRs with vector primers. Plasmid DNA was then isolated from the colonies and sequenced using pJet-specific primers. Sanger sequencing was conducted, and clear peaks in the chromatogram upstream of the PAM region indicated the presence of bi-allelic, homozygous, and/or heterozygous mutants. Sequence alignments were performed to characterize the mutation events of plants resulting in insertions, deletions or inversions.

#### Off-target analysis

To prevent potential off-target sites, the selected gRNAs were screened against the camelina genome retrieved from the EnsemblPlants database https://plants.ensembl.org/Camelina_sativa/Info/Indexusing) using the in silico tool CRISPR/Cas9 target online predictor contained in the CCTop platform (https://cctop.cos.uni-heidelberg.de/).

#### Fatty acid analyses of seeds

For fatty acid analyses, fatty acid methyl esters (FAMEs) were generated using acidic methanolysis (Miquel and Browse 1992; Ozseyhan et al. 2018; Okooboh et al. 2022). 15–20 camelina seeds were dried overnight at 105 °C and their weight recorded. 2 mL of a methanol/toluene (2:1 v/v) solution containing 2.75% (v/v) H_2_SO_4_ (95%–97%) and 2% (v/v) dimethoxypropane as well as 200 µg tripentadecanoin as internal standard were added to the seeds and incubated for 3 h at 80 °C. After cooling down, 1.5 mL 5 M NaCl and 2 mL hexane were added, samples mixed vigorously and centrifuged to achieve phase separation. The upper phase harboring the resulting FAMEs was dried down, resuspended in 500 µL acetonitrile and analyzed by gas chromatography coupled to a flame ionization detector (GC-FID) using an Agilent 6890 gas chromatograph fitted with a capillary DB-23 column (30 m × 0.25 mm, 0.25 µm coating thickness; J&W Scientific, Agilent, Santa Clara, USA). Helium was used as the carrier gas at a flow rate of 1 mL/min. The temperature gradient was first set to hold 150 °C for 1 min, then to increase from 150 to 200 °C at a rate of 4 °C/min, then from 200 to 250 °C at a rate of 5 °C/min, before being held at 250 °C for 6 min. FAMEs were identified by comparing their retention times with authentic standards.

#### Statistical data analysis

Statistical analyses were conducted using the SigmaPlot 14.0 software package (Systat Software, Inpixon, Palo Alto, CA, USA). Fatty acid proportions were evaluated using ANOVA followed by Tukey's test for post-hoc comparisons. For data that did not meet normal distribution and/or equal variance requirements, the Kruskal–Wallis test followed by the Student–Newman–Keuls method was applied. Significance levels are indicated as follows: ** for *P* ≤ 0.01, and *** for *P* ≤ 0.001.

## Supplementary Information

Below is the link to the electronic supplementary material.

Supplementary file1 (DOCX 4440 KB)

## Data Availability

All data generated during this study are included in the manuscript and Supplementary files.
