# Supplementary material for: Targeted mutagenesis of FATTY ACID ELONGASE 1 entails near complete elimination of very long chain fatty acids in the seeds of camelina cultivar Ligena
Source: aBIOTECH. 2025 Sep 30;6(4):816–30. doi: 10.1007/s42994-025-00238-z (PMC12647405; doi:10.1007/s42994-025-00238-z)
Supplement: Supplementary file 1 — Supplementary file1 (DOCX 4440 KB) [file 42994_2025_238_MOESM1_ESM.docx]

**Targeted mutagenesis of *FATTY ACID ELONGASE 1* entails near complete elimination of very long chain fatty acids in the seeds of the camelina cultivar Ligena**

Barno Ruzimurodovna Rezaeva^1^, Amélie A. Kelly^2^, Martin Fulda^2^, Ingrid Otto^1^, Iris Hoffie^1^, Sindy Chamas^1,3^, Ivo Feussner^2^, and Jochen Kumlehn^1*^

^1^ Plant Reproductive Biology, Leibniz Institute of Plant Genetics and Crop plant Research (IPK) Gatersleben, Corrensstrasse 3, 06466 Seeland, Germany

^2^ Department of Plant Biochemistry, University of Göttingen, Albrecht-von-Haller Institute and Göttingen Center for Molecular Biosciences (GZMB), Justus-von-Liebig-Weg 11, Göttingen, Germany

^3^ Research Centre for Horticultural Crops, Kühnhäuser Strasse 101, Erfurt, Germany

*Corresponding author: Jochen Kumlehn ([kumlehn@ipk-gatersleben.de](mailto:kumlehn@ipk-gatersleben.de))

**Supplementary Information**

**Contents**

**Supplementary Figures**

**Fig S1** Deep sequencing of amplicons of target regions of M_2_ siblings from the primary mutant plant CB13/19a

**Fig S2** All types of mutant sequences found in camelina protoplasts transformed with construct pBR37 carrying expression units for *cas9* and three *FAE1* target motif-specific gRNAs

**Fig S3** Deep sequencing of amplicons from target regions of four primary mutants from experiment CB13

**Fig S4** Proportions of mutated amplicon deep sequencing reads from target motifs (TM) 1, 2, and 3 in the three *FAE1* homeologs of four or five M_2_ siblings derived from each CB13/19a, 19b, 30a, and 30b

**Fig S5** Sanger sequences of amplicons from the target regions of M_2_ plants CB7/1-13, 17 and 18

**Fig S6** Relative amounts of major fatty acids (mol%) in M_3_ seed samples (15 seeds each) of cv. Ligena as wild-type control and of the 5 M_2_ plants CB7/1-13, 15, 16, 17, and 18

**Fig S7.** Total fatty acid content (µg/mg seed) in seeds of cv. Ligena (wild-type) and M_4_ seeds of triple homozygous *fae1* M_3_ mutants derived from the primary (*cas9*/gRNA/*mCHERRY*) transgenic plant CB7/1 as determined by GC-FID analysis

**Fig S8** Deep sequencing reads of off-target regions of three M_3_ siblings (plants 4, 10, and 16) derived from the M_2_ mutant plant CB7/1-18

**Supplementary Tables**

**Table S1** Comparison of different *Agrobacterium* cell densities used in transformation experiments using construct pBR38

**Table S2** Generic oligonucleotides used to amplify the target regions containing the gRNA-specific target motifs of *FAE1* without differentiation of the three homeologs

**Table S3** Homeoallele-specific oligonucleotides used to amplify the target regions containing the gRNA-specific target motifs of the three subgenomic *FAE1* variants. Resultant amplicons were used for mutation detection by Sanger sequencing

**Table S4** Oligonucleotides used for the detection of T-DNA by PCR

**Table S5** Oligonucleotides used to integrate the target-specific 5'-ends of gRNAs

**Table S6** Vector construction for targeted mutagenesis of *FAE1*

**Table S7** Composition of solutions used for protoplast isolation and transfection

**Supplementary Sequences**

**Sequences S1** Nucleotide sequences of *FAE1* homeologs in camelina cultivar Ligena

**Supplementary Data**

**Data S1** Relative amounts of major fatty acids (mol%) in seeds of cv. Ligena (wild-type) and M_4_ seeds of triple homozygous *fae1* mutants.

**Data S2** Total fatty acid content (µg/mg seed) in seeds of cv. Ligena (wild-type) and M_4_ seeds of triple homozygous *fae1* mutants.


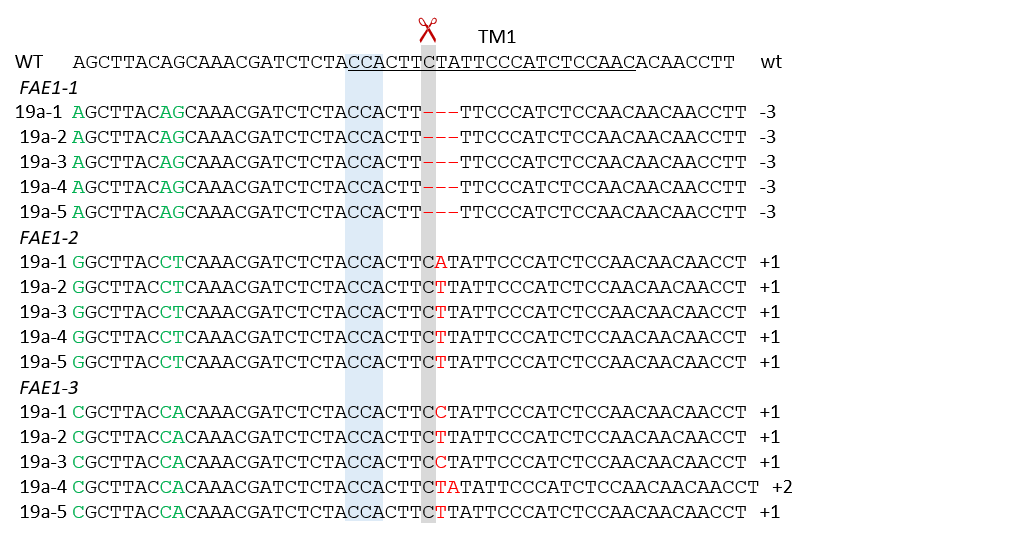

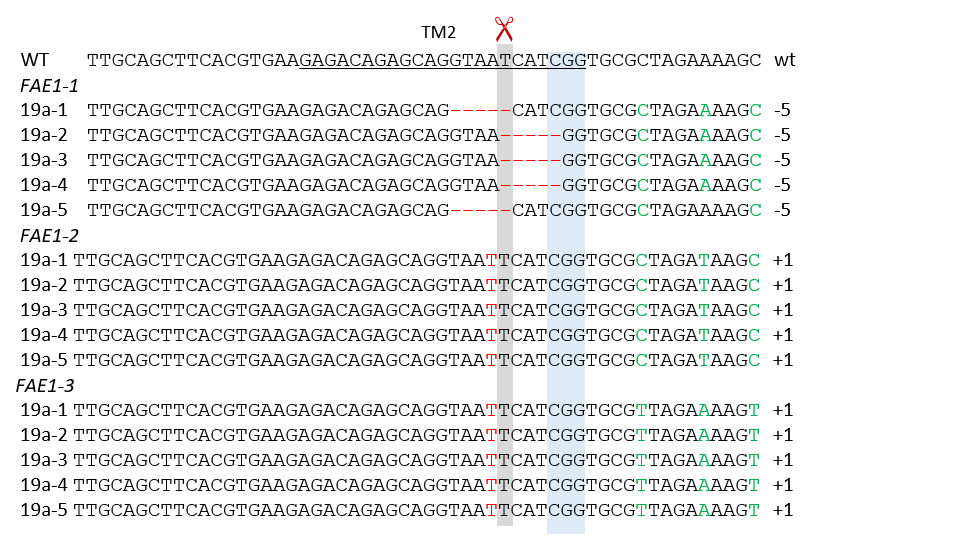


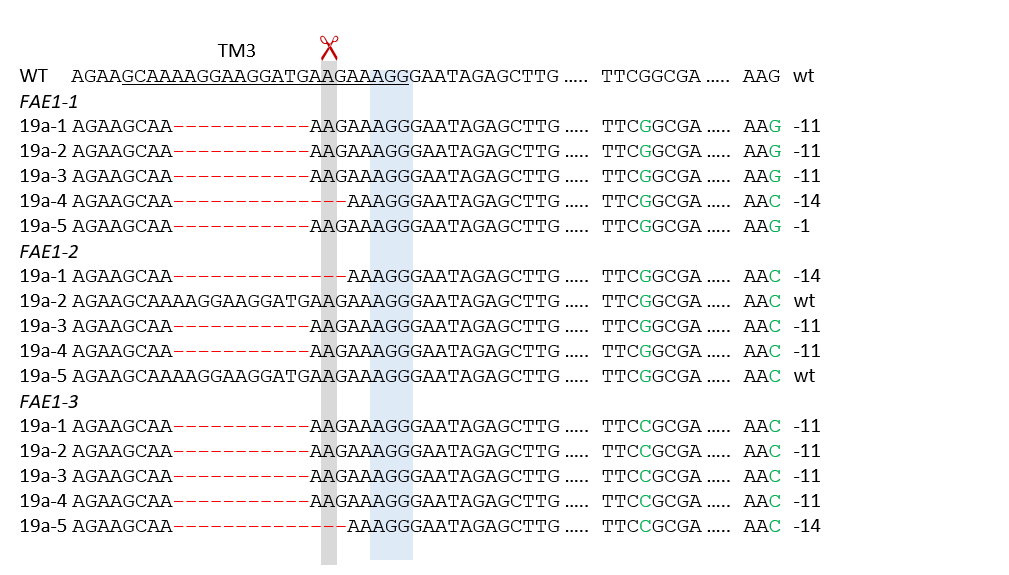


**Fig. S1** Deep sequencing of amplicons of target regions of M_2_ siblings from the primary mutant plant CB13/19a. Most frequent mutation patterns in the three target motifs of *FAE1* homeologs 1, 2, and 3. Red hyphens and letters represent deleted and inserted nucleotides, respectively. WT (*FAE1-1*) or wt indicates the respective wild-type sequence; green letters indicate nucleotide polymorphisms used to assign individual sequences to different *FAE1* homeologs. The canonical Cas9 cleavage sites are indicated by grey background. Dots denote 70 (first site) and 12 (second site) nucleotides. The target motif is underlined, with the PAM additionally indicated by light-blue background.

**
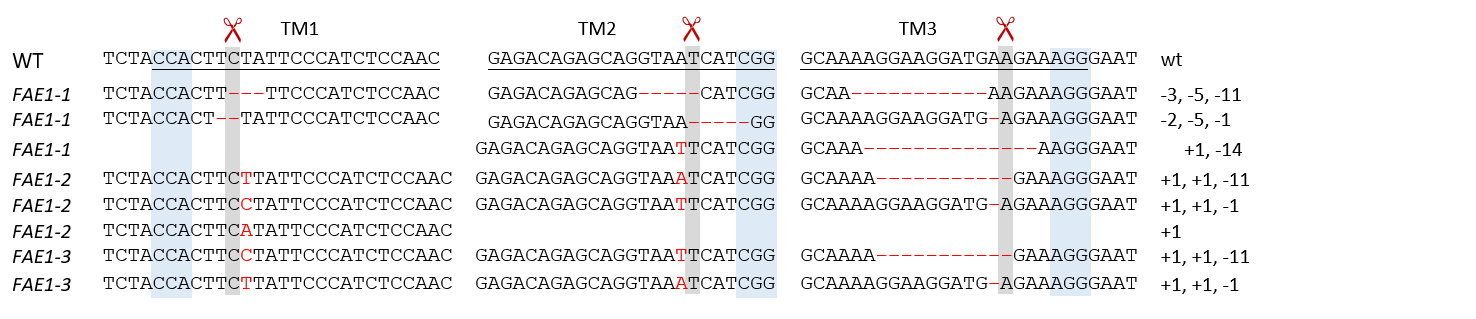
**

**Fig. S2** All types of mutant sequences found in camelina protoplasts transformed with construct pBR37 carrying expression units for *cas9* and three *FAE1* target motif-specific gRNAs. Extraction of genomic DNA and deep sequencing of amplicons of target regions was conducted two days after PEG-mediated DNA transfer. WT, wild-type sequence. Target motifs are underlined and the PAM indicated by light-blue background. Red letters and dashes indicate inserted and deleted nucleotides, respectively. Light-grey background marks the canonical DNA cleavage sites of Cas9.


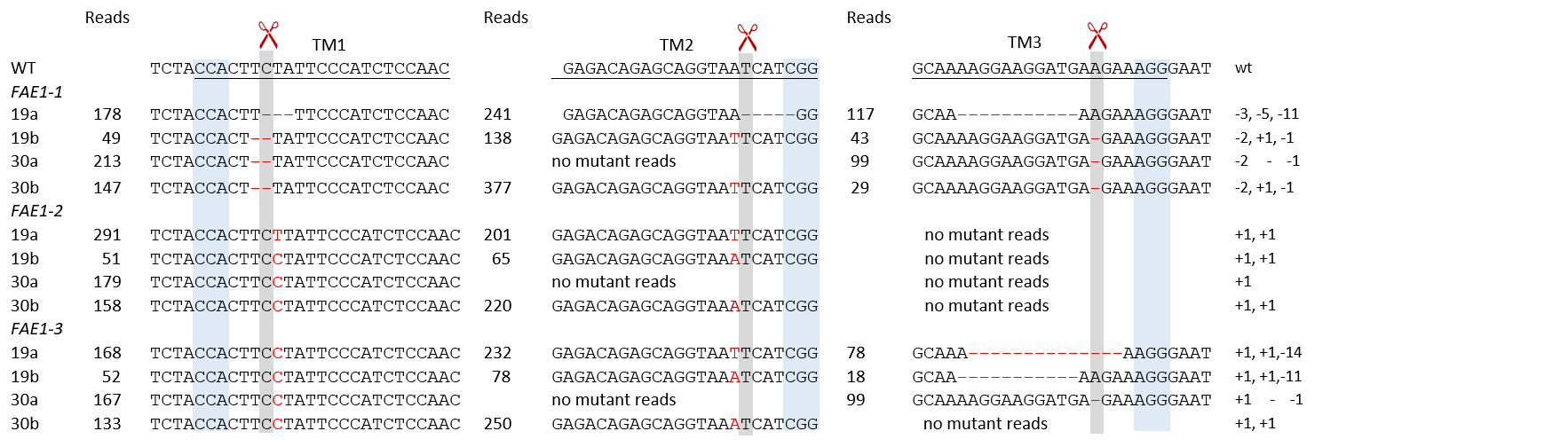


**Fig. S3** Deep sequencing of amplicons from target regions of four primary mutants from experiment CB13. Shown are the mutation patterns predominantly observed in the three target motifs of the *FAE1*-*1*, *2* and *3* homeologs. WT or wt, *FAE1-1* wild-type sequence. Target motifs with PAMs are underlined and the PAMs are highlighted by light blue background. Red letters and dashes indicate inserted or deleted nucleotides. The canonical DNA cleavage sites of Cas9 are indicated by grey background.

**
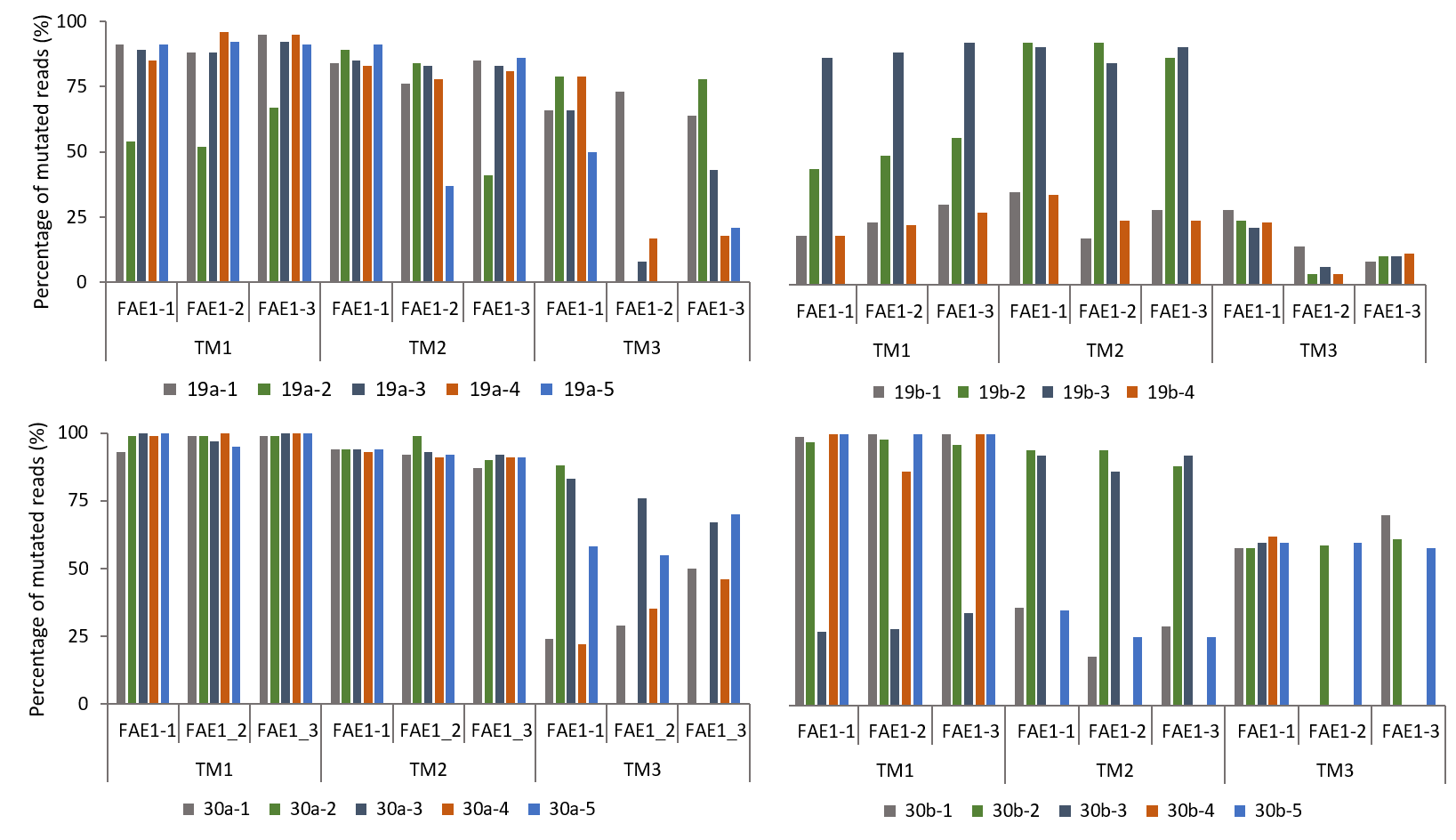
**

**Fig. S4** Proportions of mutated amplicon deep sequencing reads from target motifs (TM) 1, 2, and 3 in the three *FAE1* homeologs of four or five M_2_ siblings derived from each CB13/19a, 19b, 30a, and 30b.


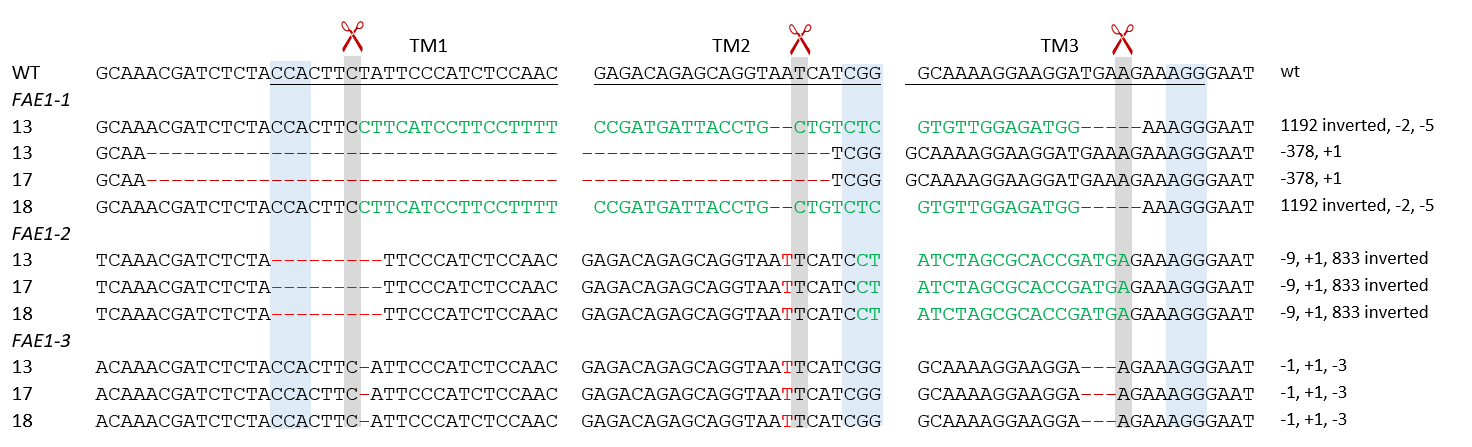


**Fig. S5** Sanger sequences of amplicons from the target regions of M_2_ plants CB7/1-13, 17 and 18. In CB7/1-13, a bi-allelic mutation was found in homeoallele *FAE1-1*, while *FAE1-2* and *FAE1-3* carried homozygous mutations were observed in *FAE1*-2 and *FAE1*-3. Moreover, the mutations in M2 plants CB7/1-17 and 18 were homozygous in all 3 homeologs and in all three target motifs. WT or wt, *FAE1-1* wild-type sequence. Target motifs are underlined and the PAMs are additionally highlighted by light-blue background. Red letters and dashes indicate inserted and deleted nucleotides. The canonical DNA cleavage sites of Cas9 are indicated by grey background. Dots denote 326 nucleotides between TM1 and TM2, and 814 nucleotides between TM2 and TM3.


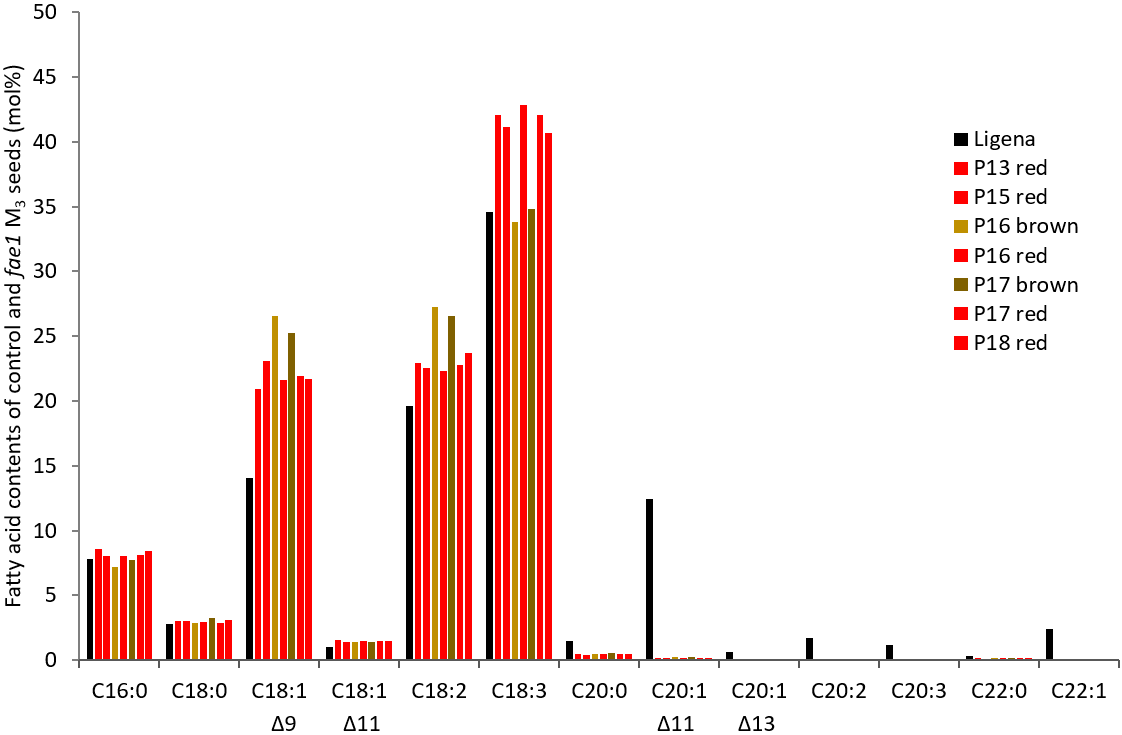


**Fig. S6** Relative amounts of major fatty acids (mol%) in M_3_ seed samples (15 seeds each) of cv. Ligena as wild-type control and of the 5 M_2_ plants CB7/1-13, 15, 16, 17, and 18. Fatty acids were measured as methyl esters by gas chromatography. CB7/1-13 contains biallelic mutations in homeolog *FAE1*-*1* and homozygous mutations in *FAE1*-2 and *FAE1*-3, while CB7/1-17 and 18 are two triple mutant plants homozygous for all three *FAE1* homeologs. Mutant patterns could not be identified for the mutant plants CBE7/1-15 and 16 in the homeologs *FAE1*-*1* and *FAE1*-2, where the amplification of target regions repeatedly failed, which indicates the presence of deletions larger than the amplified region.

**
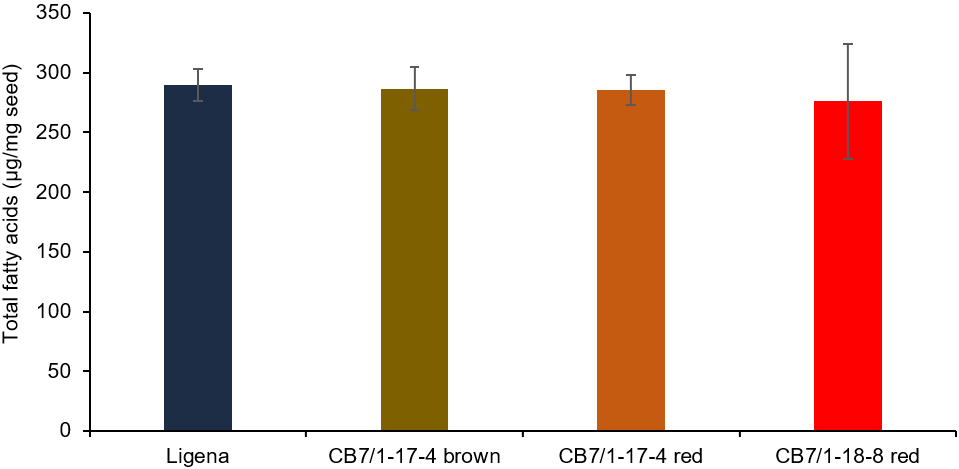
**

**Fig. S7.** Total fatty acid content (µg/mg seed) in seeds of cv. Ligena (wild-type) and M_4_ seeds of triple homozygous *fae1* M_3_ mutants derived from the primary (*cas9*/gRNA/*mCHERRY*) transgenic plant CB7/1 as determined by GC-FID analysis. Error bars denote standard deviation of six samples with 20 seeds each of Ligena wild-type, of six samples with 20 seeds each from one descendant per *fae1* M_3_ mutant. For CB7/1-17-4, two samples were analyzed: one containing mCHERRY-fluorescent seeds (transgenic) and the other containing non-fluorescent (non-transgenic) segregants. Statistical analysis (ANOVA followed by Tukey test) revealed no significant differences in total fatty acid content between the wild-type and mutants (*p* = 0.52, *p* = 0.56, *p* = 0.124).

**
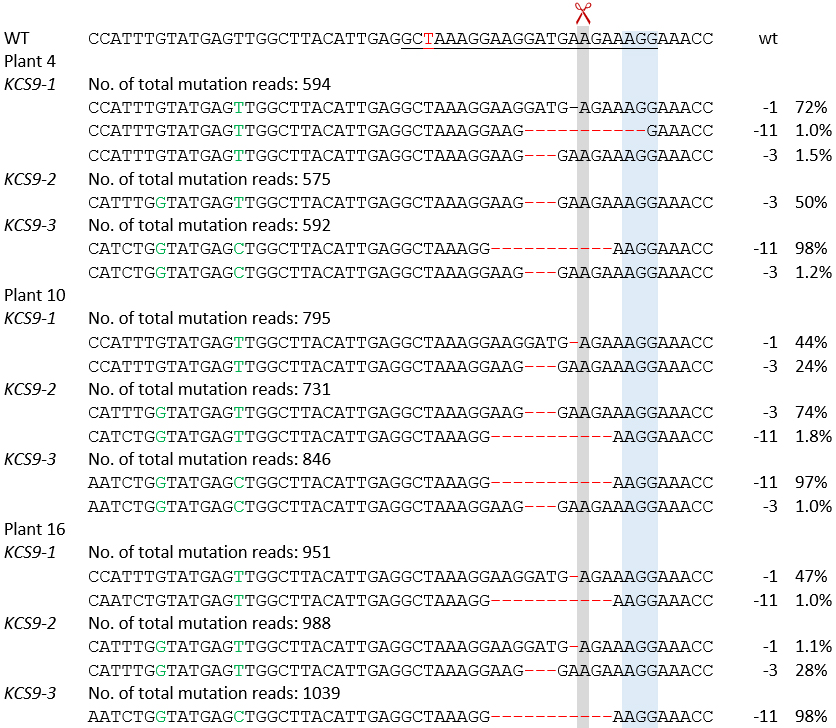
**

**Fig. S8** Deep sequencing reads of off-target regions of three M_3_ siblings (plants 4, 10, and 16) derived from the M_2_ mutant plant CB7/1-18. All types of mutation patterns found in the three target motifs of the *KCS9* homeologs 1, 2, and 3 are given, with red hyphens representing deleted nucleotides. WT or wt indicates the *KCS9-1* wild-type sequence. The red letter in the WT target motif indicates the mismatch as compared to the *FAE1* homeologs. Green letters indicate nucleotide polymorphisms used to assign individual sequences to different *KCS9* homeologs. The canonical Cas9 cleavage sites are indicated by grey background. The target motif is underlined, with the PAM additionally indicated by light-blue background.

**Table S1** Comparison of different *Agrobacterium* cell densities used in transformation experiments using construct pBR38

| Number of explants | OD_600_ variants | Number of responded explants | PCR-tested plants | *cas9*^+^ | *mCHERRY*^+^ | Number of M_1_ plants |
| --- | --- | --- | --- | --- | --- | --- |
| 60 | 0.3 | 59 | 47 | 1 | 1 | 1 |
| 60 | 0.6 | 60 | 42 | 0 | 0 | 0 |
| 60 | 0.9 | 60 | 38 | 3 | 3 | 0 |
| 60 | 0.3 | 57 | 39 | 1 | 1 | 1 |
| 60 | 0.6 | 59 | 34 | 0 | 0 | 0 |
| 60 | 0.9 | 60 | 30 | 1 | 1 | 1 |
| 60 | 0.3 | 54 | 41 | 1 | 1 | 1 |
| 60 | 0.6 | 57 | 33 | 0 | 0 | 0 |
| 60 | 0.9 | 57 | 28 | 1 | 1 | 1 |
| 60 | 0.3 | 60 | 39 | 1 | 1 | 1 |
| 60 | 0.6 | 60 | 31 | 0 | 0 | 0 |
| 60 | 0.9 | 60 | 30 | 3 | 3 | 3 |

**Table S2** Generic oligonucleotides used to amplify the target regions containing the gRNA-specific target motifs of *FAE1* without differentiation of the three homeologs. Resultant amplicons were used for mutation detection by amplicon deep sequencing, except GP400 and GP405 that were used for Sanger sequencing.

| Code | Oligo sequence 5’→3’ | Target motif | Amplicon  size (bp) |
| --- | --- | --- | --- |
| F7_Uni  R7_Uni | CAACCTTTGCTTGTTTCCG  CTAGACCTGAACGTTCTTG | 1 | 203 |
| F8_Uni  R8_Uni | CAAGAACGTTCAGGTCTAG  ACGACCATCGCAGATAGCG | 2 | 224 |
| F9_Uni  R9_Uni | GATGTGGAGGCATCTAGATC  ACCGGATATCTATCGATGC | 3 | 233 |
| F_*KCS9*  R_*KCS9* | CGAGGCATCGAGAATGACAC  CCGAAGGCTTGACATTATGC | 1 | 200 |
| F_*KCS16*  R_KCS16 | GTTGAGGCATCAAGAATGAC  AAGGATTATTAGCGGAAGGC | 1 | 200 |
| GP400  GP405 | GACGTCCGTTAACGCAAAGCTCC  GAACCGGATATCTATCGATGC | 1 + 2 + 3 | 1460 |

**Table S3** Homeoallele-specific oligonucleotides used to amplify the target regions containing the gRNA-specific target motifs of the three subgenomic *FAE1* variants. Resultant amplicons were used for mutation detection by Sanger sequencing.

| Code | Oligo sequence 5’→3’ | Target motif | Amplicon  size (bp) |
| --- | --- | --- | --- |
| F1_10  R1_10 | TGGAAAATGAAACTTGGACG  CACCACAAGTATACCAATC | 1 + 2 | 817 |
| F2_10  R2_10 | GTACAAGCTATGTCATACTG  CTTCCCAAGGACTATTCGCC | 3 | 566 |
| F3_11  R3_11 | GGTAAACGAAACTTGGACC  CCACAAGTATACCAATTTC | 1 + 2 | 805 |
| F4_11  R4_11 | GCCAGGAGATCGGAGACGG  GTTCCCAAGGACTATTCGCC | 3 | 590 |
| F5_12  R5_12 | CGAAACTTGGACCTTCAATAC  TGACCACAAGTATACCAATC | 1 + 2 | 807 |
| F6_12  R6_12 | GCTATCCAACAAGCCGGGAG  AGGACTATTCGCGGAAGCC | 3 | 595 |
| F10_10  R2_10 | GCCTCTAAGCTTACAGCAA  CTTCCCAAGGACTATTCGCC | 1 + 2 + 3 | 1342 |
| F13_11  R4_11 | AGCCTCTAGGCTTACCTCA  GTTCCCAAGGACTATTCGCC | 1 + 2 + 3 | 1343 |
| F16_12  R6_12 | CTCTACGCTTACCACAAACG  AGGACTATTCGCGGAAGCC | 1 + 2 + 3 | 1333 |

**Table S4** Oligonucleotides used for the detection of T-DNA by PCR

| Name | Oligo sequence 5’→3’ | Target gene | Amplicon size (bp) |
| --- | --- | --- | --- |
| mCHERRY_F  mCHERRY_R | GGTGCTTCACGTAGGCCTTG  CGTTCATAAGATGCCATGCC | *mCHERRY* | 489 |
| GH_ PCub_F  GH_Cas9_R | CTGAGGTGGTAGATAGTAGG  CATAGATCTGGATTACATGA | *cas9* | 568 |

**Table S5** Oligonucleotides used to integrate the target-specific 5'-ends of gRNAs. Lower case letters in the sequences indicate single-stranded overhangs after hybridization of the respective forward and reverse oligo.

| Specified gRNA | Oligo name | 5'->3' sequence |
| --- | --- | --- |
| gRNA1 | Target Motif 1_forward  Target Motif 1_ reverse | attgGTTGGAGATGGGAATAGAAG  aaacCTTCTATTCCCATCTCCAAC |
| gRNA2 | Target Motif 2_forward  Target Motif 2_ reverse | attgGAGACAGAGCAGGTAATCAT  aaacATGATTACCTGCTCTGTCTC |
| gRNA3 | Target Motif 3_forward  Target Motif 3_reverse | attgGCAAAAGGAAGGATGAAGAA  aaacTTCTTCATCCTTCCTTTTGC |

**Table S6** Vector construction for targeted mutagenesis of *FAE1*

| gRNA module | Guide RNA |  | Backbone |
| --- | --- | --- | --- |
| BR33 | 1 |  | IK75 |
| BR34 | 2 |  | IK76 |
| BR35 | 3 |  | IK77 |
| gRNA assembly module |  | Donor vectors | Backbone |
| BR36 |  | BR33, BR34, BR35 | IK61 |
| *cas9* expression module | Genetic elements | Donor vectors | Backbone |
| BR20 | PcUBI4-2-p | IK79 | IK20 |
|  | Spcas9-cds | IK81 |  |
|  | 35S-t::nos-t | IK82 |  |
| Reporter gene module | Expression unit | Donor vector | Backbone |
| BR21 | Napin-p::mCherry::E9-t | JETNapin-mCherry | BR23 |
| Transformation vector | Vector type | Donor vector | Backbone |
| BR37 | intermediate | BR36, BR20, BR21 | IK48 |
| BR38 | binary | BR37 | 6i-d35S-TE9 |

**Table S7** Composition of solutions used for protoplast isolation and transfection

| Enzyme solution | W5 solution | MMG solution | After heatbath | EG solution |
| --- | --- | --- | --- | --- |
| 1.0 % cellulase | 154 mmol/L NaCl | 4 mmol/L MES, pH 5.7 | 10 mmol/L CaCl_2_ | 40% (W/V) PEG4000 |
| 0.4% macerozyme R10 100 | 5 mmol/L KCl | 0.4 mol/L mannitol | 0.1% BSA | 100 mmol/L CaCl_2_ |
| 0.4 mol/L mannitol | 125 mmol/L CaCl_2_ | 15 mmol/L MgCl_2_ |  | 0.2 mol/L mannitol |
| 20 mmol/L KCl | 2 mmol/L MES, pH 5.7 |  |  |  |
| 20 mmol/L MES, pH 5.7 |  |  |  |  |

**Sequences S1** Sequences of the *FAE1* homeologs in camelina cultivar Ligena. Based on the sequencing data, 3 target motifs were identified for gene-specific mutagenesis of *FAE1*. The target motifs are highlighted in different colors, with the PAM sequences being additionally underlined.

Translation start: ATG

TM1: CCACTTCTATTCCCATCTCCAAC

TM2: GAGACAGAGCAGGTAATCATCGG

TM3: GCAAAAGGAAGGATGAAGAAAGG

**Ligena *CsFAE1-1ble***

GGCTAATCGACTATGGCCTTTCAGCCAATCAAATCTACGAACACGAATCCCCCTAAAACATCCTCAAGTATTTATTTAATACACATGTATCGTATTGAGCACCACTCATAAACTATTTTTTTTTTTGTTTTTAACAAAAAAAATTTATCATACTCTTTTGTAATAATAGATGCATCAACATATTGTAGGCAACGTTGAAGAACCAGTACATTCTTTTTTTTTTTGCTCCAAATTTTCAAAATTGGAAAATGAAACTTGGACGAAATAAATTTAACACTCTGTATATATTGGCAATATAATATTGCAGAGTGGACTATTTACCTTATTTTGGCAACTTTCAGTGGACTAGTAATTTATTTCAATGTGTATGCTTGCATGAGTGTGAATATACACATGTCTATATGCATGCTTGCAAATCGTAACGGACCACAAAAAAGGATCCATACAAATACCTCTTAACGGCTCCTCTCTATCATACTCTCCGACACAAACTGAGCAATGACGTCCGTTAACGCAAAGCTCCTTTACCATTACGTTCTAACCAACTTTTTCAACCTTTGCTTGTTTCCGTTAACGGCGTTACTTGCCGGAAAAGCCTCTAAGCTTACAGCAAACGATCTCTACCACTTCTATTCCCATCTCCAACACAACCTTATAACCGTAATTTTACTCTTTGCTTTCACCGCTTTCGGTTTGGTTCTCTACATTGTAACCCGGCCCAAACCGGTTTACCTCGTTGACTACTCGTGCTACCTTCCACCACCGCATCTCAAAGTTAGTGTTTCCAAGGCGATGGATATTTTCTACCAAATAAGAAAAGCTGATACCTCACGGAACGTGGCATGCGATGATCCATCCTCGCTTGATTTCCTGAGGAAGATTCAAGAACGTTCAGGTCTAGGTGATGAAACGTACAGTCCCCAGGGACTCATTAACGTGCCCCCACAAAAGACCTTTGCAGCTTCACGTGAAGAGACAGAGCAGGTAATCATCGGTGCGCTAGAAAAGCTATTCGAGAACACCAAAGTAAACCCTAGAGAGATTGGTATACTTGTGGTGAACTCAAGCATGTTTAATCCAACTCCTTCGCTATCTGCGATGGTCGTTAACACTTTCAAGCTCCGAAGCAACATCAAAAGCTTTAGTCTCGGAGGAATGGGTTGTAGTGCTGGTGTTATCGCCATTGATCTTGCAAAGGACTTGTTGCATGTTCATAAAAACACTTATGCACTTGTGGTGAGCACTGAGAACATCACTCAAGGCATTTATGCTGGCGAAAACAGATCCATGATGGTTAGCAATTGCTTGTTTCGTGTTGGTGGGGCAGCGATTTTGCTCTCCAACAAACTGGGAGATCGGAGACGGTCCAAGTACAAGCTATGTCATACTGTTCGAACGCATACCGGAGCTGATGACAAGTCTTTTCGATGTGTGCAACAAGGAGACGATGAGGGCGGTAAAATCGGAGTTTGTCTGTCAAAGGACATAACCGTTGTTGCGGGGACAGCGCTTAAGAAAAACATAGCAACGTTGGGTCCGTTGATTCTTCCTTTAAGCGAAAAGTTTCTGTTTTTAGTTACCTTCATCGCCAAGAAACTTTTGAAGGACAAGATCAAGCACTGTTACGTCCCGGATTTCAAGCTTGCTATCGACCATTTCTGTATTCATGCGGGAGGCAGAGCCGTGATCGATGTGCTTGAGAAGAGCTTAGGACTATCGCCAATCGATGTGGAGGCATCTAGATCAACGTTACATAGATTTGGGAATACTTCGTCTAGCTCAATTTGGTATGAATTGGCATACATAGAAGCAAAAGGAAGGATGAAGAAAGGGAATAGAGCTTGGCAGATTGCTTTAGGGTCAGGGTTTAAGTGTAACAGTGCGGTTTGGGTGGCTCTATGCAATGTCAAGGCTTCGGCGAATAGTCCTTGGGAAGATTGCATCGATAGATATCCGGTTCAAATTGATTCTGATTCATCAAAATCAGAGACTCATGTCAAAAACGGTCGGACCTAATTCATCTATCTGAGTGTTAACAATTTAGTGTTTTTTTCTTCTTTCCTTCTTTCTTTTAATTATTGGTTATGATTTGATAATGTTTAATGTGTTCTTTCTTTTTCGTTATAAATAAAGAAACACATGGGTGTTTCCATTCTTCTATTTAAGTTGTGGACCGTTTAATAACCTAACTTAGAATTCAATCATCTTTTTATTTTCCTTCTGTTTTTAAATCTTCCAACCAATCAACGTGTTTTCCATACTAACATAATTATTTAAAACGTGTTTAAAATTAAACATCTCTTTGGTGTTTCTAGAGGTTT

**Ligena *CsFAE1-2ble***

GGCTAATCGACTATGGCCTTTCAGCCAATCAAAGCTACGAACACGAATCTCCCTAAAACATCCTCAAGTATTTTATTTAATACACATGTATCGTATTGAGCACCACTCATAAACTAATTTCATACATTTATCATACTCTTTATTTGTAATAATAAAAGCATCAACATATTGTAGGCAATTAGAATCAAAACAAAACATTTTTTTTTTCTTTCCAAATTTTCAAAATTGGTAAACGAAACTTGGACCTTTAATACTTATATTGGCAATATAATAATATTGCAGAGTGGACTATTTCCCTTATTTTGGCAACTTTCAGTGGACTAGTAATTTATTTCAATGTGGATGCTTGCATGAGTGTGAATATACACATGTCTATATGCATGCCTGCAAATCGTAACGGACCACAAAAAAGGATCCATACAAATACCTCTTAACGGCTCCTCTCTATCATACTCTCCGACACAAACTGAGCAATGACGTCCGTTAACGCAAAGCTCCTTTACCATTACGTCCTAACCAACTTTTTCAACCTTTGCTTGTTTCCGTTAACGGCGTTACTTGCCGGAAAAGCCTCTAGGCTTACCTCAAACGATCTCTACCACTTCTATTCCCATCTCCAACACAACCTTATAACCGTAATTTTACTCTTTGCTTTCACCGCTTTCGGTTTGGTTCTCTACATTGTAACCCGGCCCAAACCGGTTTACCTCGTTGACTACTCGTGCTACCTTCCACCACCGCATCTCAAAGTTAGTGTTTCCAAGGCGATGGATATTTTCTACCAAATAAGAAAAGCTGATACCTCACGGAACGTGGCATGCGATGATCCATCCTCGCTTGATTTCCTGAGGAAGATTCAAGAACGTTCAGGTCTAGGTGATGAAACCTACAGTCCCCAGGGACTCATTAACGTGCCCCCACGAAAGACCTTTGCAGCTTCACGTGAAGAGACAGAGCAGGTAATCATCGGTGCGCTAGATAAGCTATTCGAGAATACCAAAGTTAACCCTAGAGAAATTGGTATACTTGTGGTCAACTCAAGCATGTTTAATCCAACTCCTTCGCTATCTGCGATGGTCGTTAATACTTTCAAGCTTCGAAGCAACATCAAAAGCTTTAGTCTCGGAGGAATGGGTTGTAGTGCTGGTGTCATCGCCATTGATCTTGCAAAGGACTTGTTGCATGTTCATAAAAACACTTATGCACTTGTGGTGAGCACTGAGAACATCACTCAAGGCATTTATGCTGGCGAAAATAGATCCATGATGGTTAGCAATTGCTTGTTCCGTGTTGGTGGCGCAGCGATTTTGCTCTCCAACAAGCCAGGAGATCGGAGACGGTCCAAGTACAAGTTATGTCATACTGTTCGAACGCATACCGGAGCTGATGACATGTCTTTTCGATGTGTGCAACAAGGAGACGATGAGAGCGGTAAAATCGGAGTTTGTCTGTCAAAGGACATAACCGTTGTTGCGGGGATAGCGCTTAAGAAAAACATAGCAACGTTGGGTCCGTTGATTCTTCCTTTAAGCGAAAAATTTCTGTTTTTAGTAACCTTCATCGCCAAGAAACTTTTGAAGGACAAGATCAAGCACTATTACGTCCCGGATTTCAAGCTTGCTATTGACCATTTCTGTATTCATGCGGGAGGCAGAGCCGTGATCGATGTGCTTGAGAAGAGCTTAGGACTATCTCCAATCGATGTGGAGGCATCTAGATCAACGTTACACAGATTTGGGAATACTTCGTCTAGCTCAATTTGGTATGAATTGGCATACATAGAAGCAAAAGGAAGGATGAAGAAAGGGAATAGAGCTTGGCAGATTGCTTTAGGGTCAGGATTTAAGTGTAACAGTGCGGTTTGGGTGGCTCTATGCAATGTCAAGGCTTCGGCGAATAGTCCTTGGGAACATTGCATCGATAGATATCCGGTTCAAATTGATTCTGGTTCATCAAAATCAGATACTCATGTCAAAAACGGTCGGACCTAATTCATCTATCTGAATCTGAATGTTAACCATTTAGTGTTTTTTTTTTTCCTTTCCTTC

**Ligena *CsFAE1-3ble***

AGGCTAATCGACTATGGCCTTTCAGCCAATCAAAGCTACGAACACGAATCCCCCTAAAACATCCTCAAGTATTTATTTAATACATCGTATTGAGCACCACTCATAAACTAATTCCATACATTTATCATACTGTTTATTTGTAATAATAAAAGCAGCAACATATTGTAGTTTGTAGGCAATAAGAAACAAAACAAAACATTTTTTTTTCTCTCCAAATTTTCAAAATTGGAAAACGAAACTTGGACCTTCAATACTTATATATTATATTTGCAATATAAAATTGCGAGTGGACTATTTCCCTTATTTTGGCAACTTTCAGTGGACTAGTAATTTATTTCAATGTGTATGCTTGCATGAGTGTGAATATACACATGTCTATATGCATGCCTGCAAATCGTAACGGACCACAAAAAAGGATCCATACAAATATACCTCTCAACGGCTCCTCTCTATTATGCTCTCCGACACAAACTGAGAATGACGTCCGTTAACGCAAAGCTCCTTTACCATTACGTCCTAACCAACTTTTTCAACCTTTGCTTGTTTCCGTTAACGGCGTTACTTGCCGGAAAAGCCTCTACGCTTACCACAAACGATCTCTACCACTTCTATTCCCATCTCCAACACAACCTTGTAACCGTAATTTTACTCTTTGCTTTCTCCTCTTTCGGTTTGGTTCTCTACGTTGTAACCCGGCGCAGACCGGTTTACCTCGTTGACTACTCGTGCTACCTTCCACCACCGCATCTCAAAGTTAGTGTTTCTAAGGTCATGGATATTTTCTACCAAATAAGAAAAGCTGATACCTCACGAAACGTGGCATGCGATGATCCATCCTCGCTTGATTTCCTGAGGAAGATTCAAGAACGTTCAGGTCTAGGTGATGAAACCTACAGTCCCCCGGGACTCATTCACGTGCCCCCACAAAAGACTTTTGCAGCTTCACGTGAAGAGACAGAGCAGGTAATCATCGGTGCGTTAGAAAAGTTATTCGAGAACACCAAAGTTAACCCTAGAGAGATTGGTATACTTGTGGTCAACTCAAGCATGTTTAATCCAACTCCTTCGCTATCTGCGATGGTCGTTAACACTTTCAAGCTCCGAAGCAACATCAAAAGCTTTAGTCTCGGAGGAATGGGTTGTAGTGCTGGTGTCATCGCCATTGATCTTGCAAAGGACTTGTTGCATGTTCATAAACACACTTATGCACTTGTGGTGAGCACTGAGAACATCACTCAAGGCATTTATGCTGGCGAAAATAGATCCATGATGGTTAGCAATTGCTTGTTTCGTGTTGGTGGGGCAGCGATTTTGCTATCCAACAAGCCGGGAGATCGGAGACGGTCCAAGTACAAGCTATGTCACACGGTTCGGACTCATACCGGAGCTGATGACAAGTCTTTTCGATGTGTGCAACAAGGAGACGATGAGAGCGGTAAAATCGGAGTTTGTCTGTCAAAGGACATAACAGTTGTTGCGGGGACAGCGCTTAAGAAAAACATAGCAACGTTAGGTCCGTTGATTCTTCCTTTAAGCGAAAAGTTTCTGTTCTTAGTTACCTTCATCGCCAAGAAACTTTTGAAGGACAAGATCAAGCACTATTACGTCCCGGATTTCAAGCTTGCTATTGACCATTTCTGTATTCATGCCGGAGGCAGAGCCGTAATCGATGTGCTTGAGAAGAGCTTAGGACTATCGCCAATCGATGTGGAGGCATCTAGATCAACGTTACATAGATTTGGGAATACTTCGTCTAGCTCAATTTGGTATGAATTGGCATACATAGAAGCAAAAGGAAGGATGAAGAAAGGGAATAGAGCTTGGCAGATTGCTTTAGGGTCAGGGTTTAAGTGTAACAGTGCGGTTTGGGTGGCTCTATGCAATGTCAAGGCTTCCGCGAATAGTCCTTGGGAACATTGCATCGATAGATATCCGGTTCAAATTGATTCTGATTCATCAAAATCAGAGACTCATGTCAAAAACGGTCGGACCTAATTCATCTATCTGAGTGTTAACCATTTACTGTTTTTTATTTTTCTTTCCTTCTTTCTTTTATTGGTTATGATTTGATAATGTTTAATGATGTTCTTTCTCTCTTTTTCGTTATAAACTTATATAAATAAAGAAACACATGGGTGTTTTCATTCTTCTATTTAAGTAGTGGACCATTTTATAACCTAACTTATAACTCAATCATCATTTTTTTTTTTTCTGTTTTCAACTCTTCCAACCAATCAACGTTTTTTTCATACTAACATAATTATTAAAATGTGTTTAAAATTAAACATCTCTTTGGTGTTTCTAGAGGTTT

**Data S1** Relative amounts of major fatty acids (mol%) in seeds of cv. Ligena (wild-type) and M_4_ seeds of triple homozygous *fae1* mutants as determined by GC-FID analysis. These data form the basis for Figure 2B.

| Sample | C16:0 | C18:0 | C18:1  (9Z) | C18:1  (11Z) | C18:2 | C18:3 | C20:0 | C20:1  (11Z) | C20:1  (13Z) | C20:2 | C20:3 | C22:0 | C22:1 |
| --- | --- | --- | --- | --- | --- | --- | --- | --- | --- | --- | --- | --- | --- |
| Ligena-1 | 8.13 | 2.85 | 11.64 | 1.02 | 22.07 | 34.47 | 1.93 | 10.86 | 0.73 | 1.80 | 1.28 | 0.43 | 2.78 |
| Ligena-2 | 7.74 | 2.72 | 10.37 | 1.00 | 18.75 | 37.81 | 2.03 | 11.70 | 0.78 | 1.92 | 1.59 | 0.45 | 3.13 |
| Ligena-3 | 7.98 | 2.90 | 13.49 | 1.06 | 20.93 | 34.74 | 1.92 | 10.70 | 0.73 | 1.54 | 1.12 | 0.40 | 2.47 |
| Ligena-4 | 8.37 | 3.61 | 11.45 | 0.90 | 21.84 | 32.75 | 2.57 | 11.30 | 0.73 | 1.80 | 1.18 | 0.54 | 2.97 |
| Ligena-5 | 7.45 | 3.29 | 15.09 | 1.00 | 17.71 | 33.94 | 2.39 | 12.25 | 0.80 | 1.26 | 1.08 | 0.52 | 3.22 |
| Ligena-6 | 8.37 | 3.44 | 11.26 | 1.14 | 20.83 | 34.39 | 2.53 | 10.76 | 0.98 | 1.72 | 1.19 | 0.53 | 2.87 |
| CB7/1/17brown-1 | 9.28 | 3.52 | 18.76 | 1.84 | 26.21 | 39.06 | 0.83 | 0.19 | 0.00 | 0.00 | 0.00 | 0.31 | 0.00 |
| CB7/1/17brown-2 | 8.87 | 3.26 | 20.23 | 1.68 | 25.71 | 38.92 | 0.78 | 0.20 | 0.00 | 0.04 | 0.00 | 0.30 | 0.00 |
| CB7/1/17brown-3 | 8.27 | 2.71 | 18.52 | 1.56 | 22.37 | 45.40 | 0.67 | 0.20 | 0.00 | 0.04 | 0.00 | 0.25 | 0.00 |
| CB7/1/17brown-4 | 9.25 | 3.31 | 19.10 | 1.72 | 26.95 | 38.37 | 0.80 | 0.19 | 0.00 | 0.00 | 0.00 | 0.31 | 0.00 |
| CB7/1/17brown-5 | 9.13 | 3.74 | 21.13 | 1.85 | 26.29 | 36.47 | 0.87 | 0.19 | 0.00 | 0.00 | 0.00 | 0.33 | 0.00 |
| CB7/1/17brown-6 | 9.01 | 3.12 | 18.85 | 1.81 | 25.81 | 40.10 | 0.76 | 0.20 | 0.00 | 0.05 | 0.00 | 0.29 | 0.00 |
| CB7/1/17red-1 | 8.91 | 3.33 | 18.90 | 1.65 | 24.58 | 41.28 | 0.82 | 0.20 | 0.00 | 0.00 | 0.00 | 0.32 | 0.00 |
| CB7/1/17red-2 | 8.91 | 2.73 | 20.73 | 1,47 | 25.27 | 39.64 | 0.72 | 0.22 | 0.00 | 0.00 | 0.00 | 0.30 | 0.00 |
| CB7/1/17red-3 | 8.52 | 3.04 | 18.72 | 1,42 | 22.65 | 44.35 | 0.76 | 0.21 | 0.00 | 0.04 | 0.00 | 0.30 | 0.00 |
| CB7/1/17red-4 | 9.32 | 3.15 | 20.98 | 1,57 | 27.00 | 36.62 | 0.81 | 0.21 | 0.00 | 0.00 | 0.00 | 0.33 | 0.00 |
| CB7/1/17red-5 | 8.74 | 2.96 | 18.69 | 1,48 | 23.04 | 43.77 | 0.75 | 0.21 | 0.00 | 0.04 | 0.00 | 0.30 | 0.00 |
| CB7/1/17red-6 | 8.53 | 3.01 | 19.47 | 1,60 | 23.77 | 42.36 | 0.73 | 0.21 | 0.00 | 0.04 | 0.00 | 0.28 | 0.00 |
| CB7/1/18/4red-1 | 9.26 | 3.84 | 19.41 | 1,66 | 25.93 | 39.05 | 0.51 | 0.17 | 0.00 | 0.00 | 0.00 | 0.17 | 0.00 |
| CB7/1/18/4red-2 | 9.76 | 3.52 | 19.15 | 1,68 | 28.96 | 36.08 | 0.50 | 0.17 | 0.00 | 0.00 | 0.00 | 0.17 | 0.00 |
| CB7/1/18/4red-3 | 9.36 | 3.46 | 19.58 | 1,51 | 25.67 | 39.03 | 0.82 | 0.20 | 0.06 | 0.00 | 0.00 | 0.31 | 0.00 |
| CB7/1/18/4red-4 | 9.37 | 3.17 | 19.47 | 1,66 | 26.47 | 38.83 | 0.61 | 0.19 | 0.00 | 0.00 | 0.00 | 0.23 | 0.00 |
| CB7/1/18/4red-5 | 8.61 | 3.69 | 19.69 | 1,62 | 22.74 | 42.83 | 0.47 | 0.18 | 0.00 | 0.00 | 0.00 | 0.16 | 0.00 |
| CB7/1/18/4red-6 | 8.85 | 2.98 | 19.32 | 1,55 | 24.28 | 41.75 | 0.73 | 0.21 | 0.00 | 0.04 | 0.00 | 0.28 | 0.00 |

**Data S2** Total fatty acid content (µg/mg seed) in seeds of cv. Ligena (wild-type) and M_4_ seeds of triple homozygous *fae1* mutants derived from the primary (*cas9*/gRNA/*mCHERRY*) transgenic plant CB7/1 as determined by GC-FID analysis. These data form the basis for Figure S7.

| Replicate | Sample | Total fatty acids |
| --- | --- | --- |
| Ligena-1 | Ligena | 289.48 |
| Ligena-2 | Ligena | 288.22 |
| Ligena-3 | Ligena | 285.62 |
| Ligena-4 | Ligena | 288.91 |
| Ligena-5 | Ligena | 289.29 |
| Ligena-6 | Ligena | 286.95 |
| CB7/1/17brown-1 | CB7/1/17 | 286.49 |
| CB7/1/17brown-2 | CB7/1/17 | 284.01 |
| CB7/1/17brown-3 | CB7/1/17 | 283.37 |
| CB7/1/17brown-4 | CB7/1/17 | 280.59 |
| CB7/1/17brown-5 | CB7/1/17 | 282.73 |
| CB7/1/17brown-6 | CB7/1/17 | 284.32 |
| CB7/1/17red-1 | CB7/1/17 | 285.71 |
| CB7/1/17red-2 | CB7/1/17 | 289.38 |
| CB7/1/17red-3 | CB7/1/17 | 292.09 |
| CB7/1/17red-4 | CB7/1/17 | 292.42 |
| CB7/1/17red-5 | CB7/1/17 | 292.46 |
| CB7/1/17red-6 | CB7/1/17 | 294.30 |
| CB7/1/18/4red-1 | CB7/1/18/4red | 275.93 |
| CB7/1/18/8red-2 | CB7/1/18/4red | 274.01 |
| CB7/1/18/8red-3 | CB7/1/18/4red | 275.42 |
| CB7/1/18/8red-4 | CB7/1/18/4red | 271.25 |
| CB7/1/18/8red-5 | CB7/1/18/4red | 314.08 |
| CB7/1/18/8red-6 | CB7/1/18/4red | 303.62 |
